# Novel Biological Functions of the NsdC Transcription Factor in Aspergillus fumigatus

**DOI:** 10.1128/mBio.03102-20

**Published:** 2021-01-05

**Authors:** Patrícia Alves de Castro, Clara Valero, Jéssica Chiaratto, Ana Cristina Colabardini, Lakhansing Pardeshi, Lilian Pereira Silva, Fausto Almeida, Marina Campos Rocha, Roberto Nascimento Silva, Iran Malavazi, Wenyue Du, Paul S. Dyer, Matthias Brock, Flávio Vieira Loures, Koon Ho Wong, Gustavo H. Goldman

**Affiliations:** a Faculdade de Ciências Farmacêuticas de Ribeirão Preto, Universidade de São Paulo, Ribeirão Preto, Brazil; b Faculty of Health Sciences, University of Macau, Taipa, Macau SAR, China; c Genomics and Bioinformatics Core, Faculty of Health Sciences, University of Macau, Taipa, Macau SAR, China; d Faculdade de Medicina de Ribeirão Preto, Universidade de São Paulo, Ribeirão Preto, Brazil; e Departamento de Genética e Evolução, Centro de Ciências Biológicas e da Saúde, Universidade Federal de São Carlos, São Carlos, São Paulo, Brazil; f Fungal Genetics and Biology Group, School of Life Sciences, University of Nottingham, Nottingham, United Kingdom; g Instituto de Ciência e Tecnologia, Universidade Federal de São Paulo, São José dos Campos, Brazil; h Institute of Translational Medicine, Faculty of Health Sciences, University of Macau, Taipa, Macau SAR, China; Duke University Medical Center

**Keywords:** *Aspergillus fumigatus*, sexual cycle, transcription factor, cell wall integrity pathway, calcium signaling, transcription factors

## Abstract

The fungal zinc finger transcription factor NsdC is named after, and is best known for, its essential role in sexual reproduction (never in sexual development). In previous studies with Aspergillus nidulans, it was also shown to have roles in promotion of vegetative growth and suppression of asexual conidiation. In this study, the function of the *nsdC* homologue in the opportunistic human pathogen A. fumigatus was investigated. NsdC was again found to be essential for sexual development, with deletion of the *nsdC* gene in both *MAT1-1* and *MAT1-2* mating partners of a cross leading to complete loss of fertility. However, a functional copy of *nsdC* in one mating partner was sufficient to allow sexual reproduction. Deletion of *nsdC* also led to decreased vegetative growth and allowed conidiation in liquid cultures, again consistent with previous findings. However, NsdC in A. fumigatus was shown to have additional biological functions including response to calcium stress, correct organization of cell wall structure, and response to the cell wall stressors. Furthermore, virulence and host immune recognition were affected. Gene expression studies involving chromatin immunoprecipitation (ChIP) of RNA polymerase II (PolII) coupled to next-generation sequencing (Seq) revealed that deletion of *nsdC* resulted in changes in expression of over 620 genes under basal growth conditions. This demonstrated that this transcription factor mediates the activity of a wide variety of signaling and metabolic pathways and indicates that despite the naming of the gene, the promotion of sexual reproduction is just one among multiple roles of NsdC.

## INTRODUCTION

Aspergillus fumigatus is an opportunistic human fungal pathogen and the main causal agent of invasive aspergillosis, a life-threatening infection especially in immunocompromised patients (1). It is estimated that 300,000 infections occur per year worldwide with an associated mortality that could reach 95% (2, 3). The fungus is ubiquitous in the environment, and the infection is acquired mainly by inhalation of conidia present in the air (4). These conidia are produced on structures called conidiophores which are characteristic of asexual reproduction in *Aspergillus* species (5, 6). A. fumigatus can undergo both asexual and sexual reproductive cycles, but whereas asexual reproduction is observed in almost all aspergilli, sexual reproduction has been detected only in approximately 36% of *Aspergillus* species (7, 8).

The regulation of asexual reproduction involves several genes and pathways in A. fumigatus. A highly conserved pathway has been described as the central regulatory mechanism of asexual development in *Aspergillus* species such as A. fumigatus (6). It involves three key regulators that behave as a cascade activating each other in sequential order: BrlA, a transcription factor (TF) which is an essential activator; AbaA, responsible for hyphal differentiation into phialides; and WetA, important for conidial maturation (9–13). Accumulation of mRNA of the *abaA* and *wetA* genes was detected during asexual reproduction of A. fumigatus, while deletion of *brlA* impaired *abaA* and *wetA* transcript production (12). Further complementary regulatory mechanisms have also been reported to play a role in the genetic control of conidiation, such as the velvet family of negative regulator proteins (14), the FluG and FlbA-E upstream activators (11, 15), and members of the heterotrimeric G-protein signaling pathway (6, 11, 16, 17). Moreover, we have recently found that the mitogen-activated protein kinase MpkB is also important for conidiation and melanin production in A. fumigatus (18).

Despite evidence for cryptic sexual development in A. fumigatus having already been reported in the literature (19–21), it was not until 2009 that O’Gorman and colleagues were able to demonstrate the induction of a heterothallic (obligate outbreeding) sexual cycle under well-defined laboratory conditions (22). This involved formation of sexual fruiting bodies known as cleistothecia which at maturity contained sexual ascospores, with evidence provided for genetic recombination in the offspring. Sexual reproduction was found to require very specific environmental conditions not easily found in nature and incubation for up to 12 months. However, the subsequent identification of “supermater” strains (with enhanced sexual fertility) has facilitated the investigation of this process in a laboratory setting (23). The sexual cycle has provided an excellent tool not just for expanding basic genetic studies but also for investigating and explaining medically important processes such as the acquisition of azole resistance or differences in virulence in strains of A. fumigatus (24). The breeding system in A. fumigatus is governed by the presence of two mating-type (*MAT*) genes (*MAT1-1* and *MAT1-2*) which encode alpha- and high-mobility group (HMG) domain transcription factors, respectively, and determine sexual identity of isolates (6, 20). Deletion of either of these genes resulted in a block in cleistothecium formation (25). Distribution of the compatible mating types has been reported to be near 1:1 in both regional and global populations of the fungus (20, 26).

Although a sexual cycle has only recently been described in A. fumigatus, Aspergillus nidulans has long been established as a model organism for studying processes controlling sexual development in ascomycete fungi. Beyond *MAT* genes, a wide list of over 70 sex-related genes has also been identified in A. nidulans, including genes with roles in light and nutrient sensing, signal transduction pathways, genes encoding transcription factors and other regulatory proteins, or even genes linked to endogenous physiological processes (27). Some of these findings have been confirmed to apply to A. fumigatus, such as the involvement of the transcription factor NsdD in hyphal fusion, necessary for heterokaryon formation (25). However, it is important to note that whereas A. fumigatus is a heterothallic species, in contrast A. nidulans is a homothallic (self-fertile) species and there is limited evidence that the regulation of sexual reproduction may differ slightly in homothallic versus heterothallic species (28).

The NsdC (never in sexual development) transcription factor was first identified in A. nidulans specifically due to its requirement for sexual reproduction. It encodes a fungus-specific C_2_H_2_-type zinc finger transcription factor, and its loss resulted in the lack of fruiting body formation, retarded vegetative growth, and hyperactive asexual sporulation (29). In the present work, we have characterized the homologous NsdC transcription factor from A. fumigatus, which was initially identified in a screen of transcription factor null mutants showing sensitivity when exposed to high concentrations of calcium (30). Besides calcium, we now report that the Δ*nsdC* strain exhibits increased sensitivity to cell wall-damaging agents. Furthermore, deletion of *nsdC* in both mating partners was found to completely abolish sexual development, while asexual conidiation was derepressed in liquid medium. Transmission electron microscopy, cell wall staining studies, and cell wall sugar content measurements showed that NsdC is involved in cell wall organization and composition, while genome-wide transcription profiling analysis (as measured by RNA polymerase II [PolII] occupancy) revealed NsdC has roles in several important biological responses. Finally, the Δ*nsdC* strain displayed a reduction in mortality and fungal burden in a neutropenic mouse model of invasive aspergillosis coupled with enhanced killing by macrophages. In summary, it is concluded that NsdC is important not only for sexual and asexual development but also for calcium tolerance and cell wall damage stress response by affecting cell wall composition and organization and has a role in virulence of A. fumigatus.

## RESULTS

### NsdC is important for calcium tolerance.

**FIG 1:**
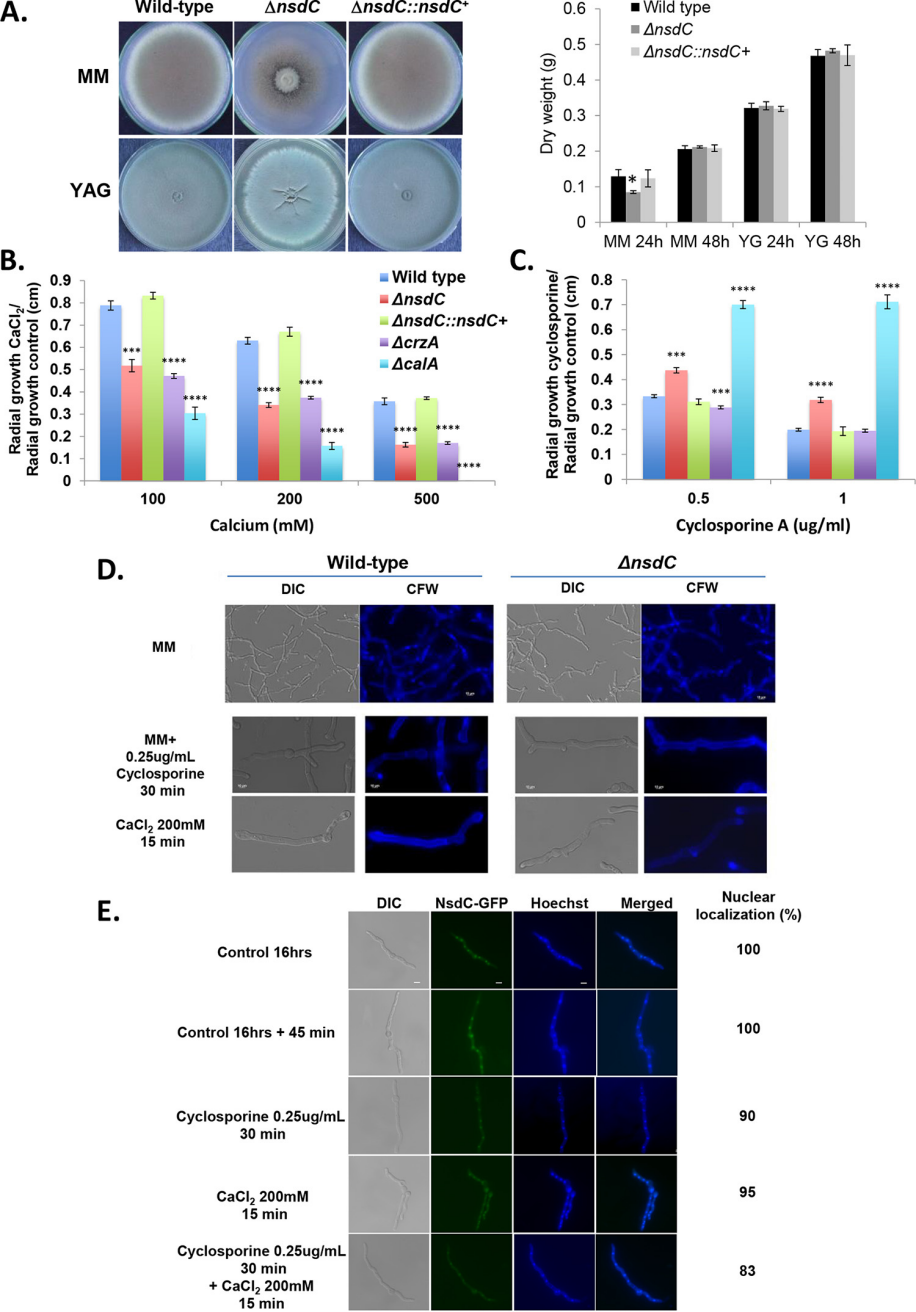
Growth phenotypes for Δ*nsdC*. (A to C) The wild-type, Δ*nsdC*, and Δ*nsdC*::*nsdC*^+^ strains were grown for 5 days on solid MM and YAG or for 2 days in liquid MM and YG at 37°C (A), MM + CaCl_2_ (B), and MM + cyclosporine (C). Results are expressed as radial growth of treatment/radial growth of control (centimeters) and are the average of three independent biological repetitions ± standard deviation. The statistical analysis was one-tailed, paired *t* test. *P* values: *, *P* < 0.05; **, *P* < 0.01; ***, *P* < 0.001; ****, *P* < 0.0001. (D) Conidia of the wild-type and Δ*nsdC* strains were germinated for 16 h at 37°C in liquid MM and exposed or not to 0.25 μg/ml cyclosporine for 30 min or 200 mM CaCl_2_ for 15 min. Germlings were stained with calcofluor white (CFW). DIC, differential interference contrast. First row shows ×40 magnification while second and third rows show ×100 magnification. (E) NsdC-GFP germlings were grown for 16 h at 37°C in MM and exposed to 200 mM CaCl_2_ for 15 min or 0.25 μg/ml cyclosporine for 30 min followed by 200 mM CaCl_2_ for 15 min. About 100 germlings were counted in each treatment. Bar, 5 μm. Statistical analysis was performed using a one-way ANOVA comparing both Δ*nsdC* and Δ*nsdC*::*nsdC*^+^ strains to the wild type (****, *P* < 0.0001).

We previously identified, in a library of 395 A. fumigatus TF null mutants, nine null mutants that exhibited increased sensitivity to 500 mM calcium chloride (30). Among these TF mutants, we observed Δ*nsdC* (Afu7g03910), a putative homologue of A. nidulans NsdC (AN4263), a C_2_H_2_ zinc finger TF required for sexual development (29). In order to investigate the function of *nsdC* in A. fumigatus, the null mutant strain was here complemented by reinsertion of the wild-type gene and aspects of growth were compared between the deletion mutant and the complemented strain. The deletion of *nsdC* in A. fumigatus affected about 30 and 10% radial growth reduction on solid minimal medium (MM) and complete medium (yeast agar glucose [YAG]), respectively (Fig. 1A). There is about 20% growth reduction in liquid MM in 24-h growth compared to the wild type, but at 48 h growth is comparable to both wild-type and complemented strains (Fig. 1A, right panel). Interestingly, there are no growth differences in liquid YG medium for all three strains (Fig. 1A, right panel). The Δ*nsdC* mutant was more sensitive to calcium (CaCl_2_) (Fig. 1B) and exhibited enhanced resistance to the calcineurin activity inhibitor cyclosporine (Fig. 1C). We included these experiments with cyclosporine to see the relationship between calcineurin and NsdC. The Δ*calA* mutant (null mutant for the calcineurin catalytic subunit) is also sensitive to calcium but resistant to cyclosporine (Fig. 1B and C). The Δ*crzA* mutant is sensitive to calcium but as sensitive to cyclosporine as the wild-type strain (Fig. 1B and C). We have not observed differences between the morphology and growth of the wild-type and Δ*nsdC* germlings (Fig. 1D). NsdC-GFP (green fluorescent protein) under the control of the *nsdC* promoter (the cassette was homologously integrated in the *nsdC* locus) was constitutively present in the nucleus, and there was no clear effect on its translocation to the cytoplasm and/or degradation after exposure to high concentrations of CaCl_2_ or cyclosporine (Fig. 1E). Taken together, these results strongly suggest that NsdC is involved in the A. fumigatus calcium response pathway.

### Generation of Δ*nsdC* mutants in high-fertility *MAT1-1* and *MAT1-2* mating-type backgrounds.

In the homothallic fungus Aspergillus nidulans, the *nsdC* gene is crucial for sexual development (29). Therefore, it was of interest to investigate whether the same is true for the heterothallic fungus A. fumigatus. Unfortunately, the *MAT1-1* wild-type strain CEA17 (and its KU^80^ mutant and derivatives used elsewhere in the present study) was found to be sterile in preliminary crossing efforts, which made it difficult to assess the specific contribution of *nsdC* to sexual development in this background. To overcome this limitation, the *nsdC* gene was instead deleted in the high-fertility *MAT1-1* wild-type strain 47-51 (AfIR974 [22]). As expected, some of the transformants displayed a condensed growth phenotype and green pigmentation of the mycelium on the regeneration plates, consistent with the phenotype observed with the Δ*nsdC* mutation in the CEA17 background (Fig. 1A), and *nsdC* deletion was confirmed in these transformants.

**FIG 2:**
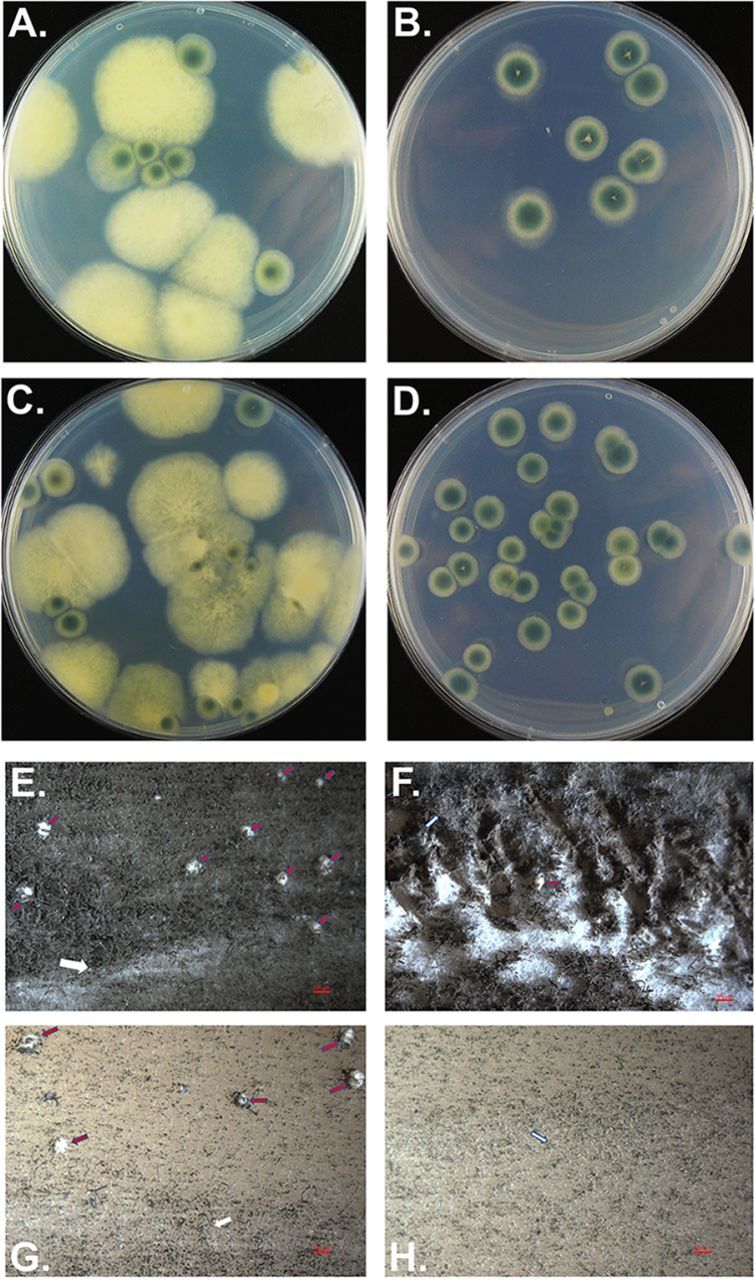
Sexual crosses of the Δ*nsdC* mutant. Appearance of sexual progeny from crosses using 47-51Δ*nsdC* (5) as a mating partner, in which *nsdC* was deleted by replacement with a pyrithiamine resistance cassette. (A and B) Offspring of the cross 47-51Δ*nsdC* (5) × 47-55. (C and D) Offspring of the cross 47-51 Δ*nsdC* (5) × 47-107. Ascospore progeny were spread plated onto either Glucose 50, Gln 10 (A and C) or Glucose 50, Gln 10, PT (pyrithiamine) 0.1 (B and D) medium; see Materials and Methods for details. All pyrithiamine-resistant (at 0.1 μg/ml PT) offspring (B and C) show the Δ*nsdC* phenotype. (E to H) Cleistothecia formed by representative mating partners of A. fumigatus on oatmeal agar following incubation for 4 months in darkness at 30°C. Images photographed after “hoovering” of plates as appropriate to allow clearer visualization of cleistothecia. Red arrows indicate cleistothecia, and white arrows indicate the barrage zone where two mating types of A. fumigatus strains met. Bar, 500 μm. (E) Cleistothecia formed in a cross between strains 47-267 (*MAT1-1*) and 47-55 (*MAT1-2*). (F) Cleistothecia formed in a cross between 47-267 and Δ*nsdC*-a (*MAT1-2*). (G) Cleistothecia formed by 47-55 and Δ*nsdC-*b (*MAT1-1*). (H) Absence of cleistothecia in a cross between Δ*nsdC-*a and Δ*nsdC*-b.

To test the fertility of Δ*nsdC* mutants with the 47-51 parental background, and to potentially generate Δ*nsdC* mutants with a *MAT1-2* mating-type background, crosses were set up between the known fertile A. fumigatus *MAT1-2* strains 47-55 and 47-107 and two representative Δ*nsdC MAT1-1* mutants, 47-51Δ*nsdC4* and 47-51Δ*nsdC5* (see Table S1 at https://doi.org/10.6084/m9.figshare.12931754.v3). All crosses were found to produce cleistothecia and viable ascospores (see Table S2 at https://doi.org/10.6084/m9.figshare.12931754.v3). This clearly demonstrated that *nsdC* is not essential for fertility in crosses with a heterothallic wild-type mating partner, at least when *nsdC* is deleted in the *MAT1-1* genetic background. The Δ*nsdC* mutants appeared to show slightly lower fertility than the 47-51 parent when crossed to isolate 47-55, but this reduction was not statistically significant (*F* = 1.369; *P* value = 0.2820). When ascospores were plated on a medium without pyrithiamine, both wild-type and Δ*nsdC* mutant phenotypes were observed, whereas only the Δ*nsdC* phenotype occurred on plates with pyrithiamine (Fig. 2A to D). This was consistent with the use of the pyrithiamine resistance cassette in *nsdC* deletion. Ascospore offspring from crosses between 47-51Δ*nsdC5* and 47-107 and between 47-51Δ*nsdC5* and 47-55 were then randomly selected from the pyrithiamine plate and analyzed for their mating type (Fig. 2A to D). These Δ*nsdC* progeny showed an approximate 1:1 ratio of *MAT1-1* to *MAT1-2* genotypes, confirming that sexual recombination had occurred as demonstrated by the appearance of the novel Δ*nsdC MAT1-2* genotypes (Fig. 2A to D; see also Table S1 at https://doi.org/10.6084/m9.figshare.12931754.v3).

PCR analysis of these transformants further confirmed the successful modification of the *nsdC* gene in these transformants (see Fig. S1 at https://doi.org/10.6084/m9.figshare.12931754.v3).

### Impact of both *MAT1-1* and *MAT1-2 nsdC* deletion on sexual crossing of A. fumigatus.

Since a *MAT1-1* Δ*nsdC* strain was fertile when crossed with a highly fertile *MAT1-2* strain, we were interested to see whether this also held true for a Δ*nsdC* mutant with a *MAT1-2* mating-type background. Furthermore, we investigated the result of crossing of eight additional Δ*nsdC* strains, comprised of two *MAT1-1* and two *MAT1-2* strains from progeny of each of crosses between 47-51-Δ*nsdC*5 and 47-55 or 47-51-Δ*nsdC5* and 47-107 (see Table S2 at https://doi.org/10.6084/m9.figshare.12931754.v3).

As shown in Table 1 and Fig. 2E to H, cleistothecia were formed in all control crosses between *MAT1-1* and *MAT1-2* wild-type isolates, and also in almost all crosses when at least one mating partner contained a functional copy of the *nsdC* gene, irrespective of whether it was a *MAT1-1* or *MAT1-2* partner that contained the functional copy. The only exceptions were crosses 47-267 × Δ*nsdC*-w and 47-107 × Δ*nsdC*-y, which failed to form cleistothecia, but importantly strains Δ*nsdC*-w and Δ*nsdC*-y formed cleistothecia with the alternative mating partners 47-51 and 47-55, respectively. In contrast, deletion of *nsdC* in both mating partners resulted in a complete lack of the formation of cleistothecia in all 16 test crosses, despite incubation for an extended period of 6 months and the formation of a barrage zone between the partners (Table 1; Fig. 2E to H). Therefore, it can be concluded that whereas gene activation by *nsdC* in one mating partner is sufficient to initiate and allow completion of the sexual cycle, the absence of *nsdC* in both mating partners instead totally abolishes sexual development.

**TABLE 1:** Formation of cleistothecia in crosses between various *MAT1-1* A. fumigatus wild-type and Δ*nsdC* strains and various *MAT1-2* A. fumigatus wild-type and Δ*nsdC* strains^*a*^

*MAT1-2* strain	*MAT1-1* strain					
	47-51	47-267	Δ*nsdC*-b	Δ*nsdC*-z	Δ*nsdC*-y	Δ*nsdC*-d
47-55	7.0 ± 5.7	29.7 ± 8.0	13.8 ± 9.2	2.8 ± 2.1	7.8 ± 7.6	8.3 ± 11.0
47-107	14.2 ± 8.7	0.3 ± 0.5	4.0 ± 4.7	0.5 ± 0.6	0	0.5 ± 1.0
Δ*nsdC*-a	0.5 ± 1.0	1.5 ± 1.7	0	0	0	0
Δ*nsdC*-c	5.5 ± 5.9	2.7 ± 5.5	0	0	0	0
Δ*nsdC*-x	35.0 ± 21.8	21.2 ± 8.1	0	0	0	0
Δ*nsdC*-w	40.7 ± 18.9	0	0	0	0	0

a Numbers indicate average numbers of cleistothecia per 9-cm crossing plate ± SEM.

### NsdC is involved in the control of asexual sporulation.

**FIG 3:**
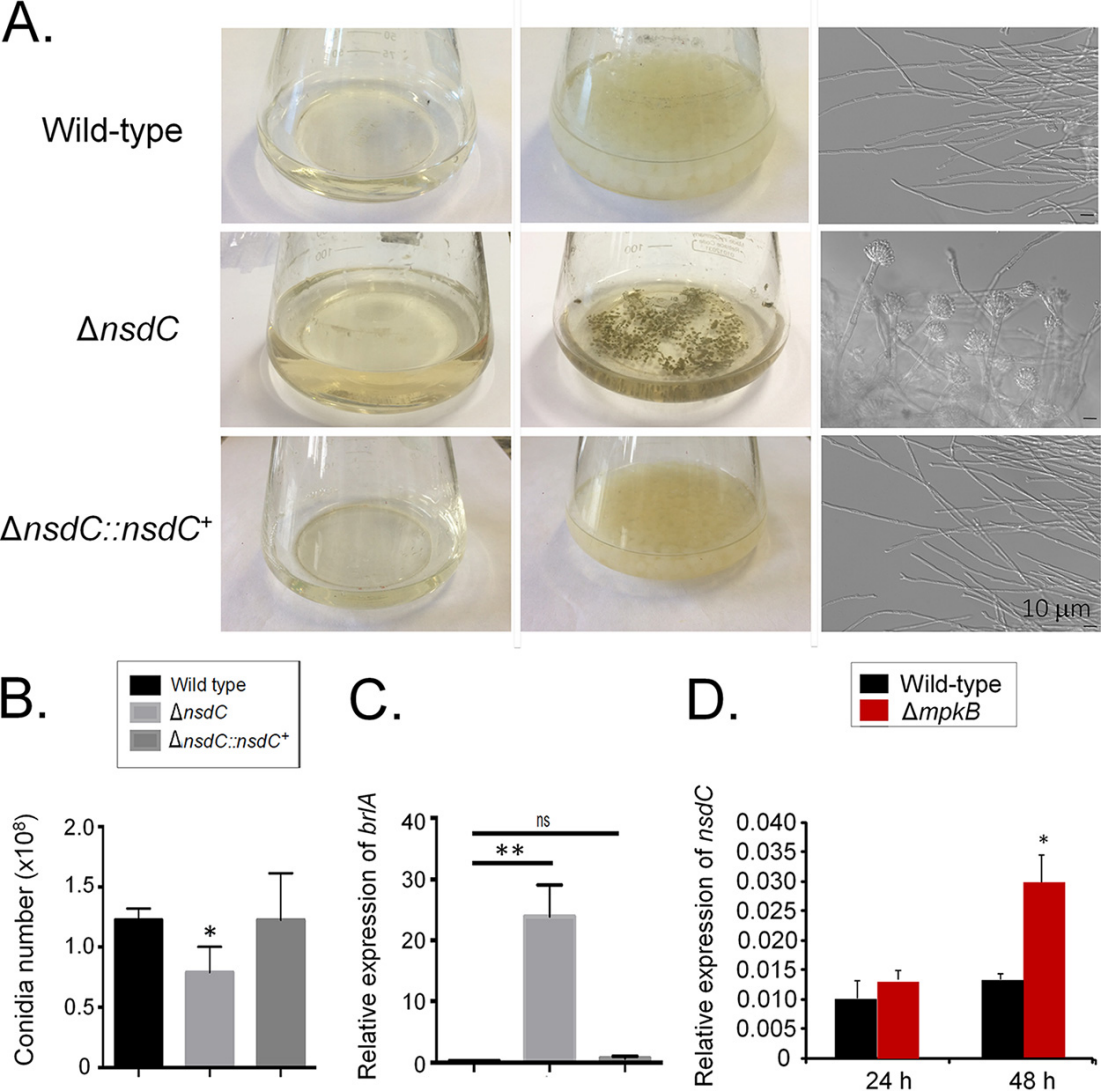
The A. fumigatus *nsdC* null mutant produces conidiophores in liquid cultures with agitation. (A) Cultures (center column) and separated supernatants from the same cultures (left column) were grown with agitation for 5 days at 37°C. DIC microscopic analysis revealed the presence of conidia and conidiophores in the Δ*nsdC* cultures (right column). (B) Number of conidia in the wild-type, Δ*nsdC*, and Δ*nsdC*::*nsdC^+^* strains grown for 5 days at 37°C on MM. (C) The wild-type, Δ*nsdC*, and Δ*nsdC*::*nsdC^+^* strains were grown for 24 h at 37°C in MM. Gene expression was obtained for *brlA* and was normalized by using *tubA* (Afu1g10910). (D) The wild-type and Δ*mpkB* strains were grown for 24 or 48 h at 37°C in MM. Gene expression was obtained for *nsdC* and was normalized by using *tubA* (Afu1g10910). Error bars represent standard deviations of the average of three independent biological repetitions (each with 2 technical repetitions). Statistical analysis was performed using a one-way ANOVA compared to the wild-type condition (*, *P* < 0.05; **, *P* < 0.01; ns, not significant).

The Δ*nsdC* strain displayed deregulation in initiation of conidiation in liquid MM, with conidiophores and conidia being produced, unlike the control wild-type parent and complemented strains (Fig. 3A). However, a significant reduction in the number of conidia produced on solid MM by the Δ*nsdC* strain was observed in comparison to the control strains (Fig. 3B). Previous reports described that the transcription factor BrlA is important for the activation of conidial development in A. fumigatus (11). RT-qPCR experiments showed that *brlA* expression in the Δ*nsdC* strain was 25 times higher in liquid MM than in the wild-type and complemented strains (Fig. 3C). We have previously observed that the mitogen-activated protein (MAP) kinase MpkB plays an important role in the negative control of conidiation in liquid medium (18). The accumulation of *nsdC* mRNA was dependent on MpkB since there was a 2-fold increase in the *nsdC* mRNA transcripts in the Δ*mpkB* strain compared with the wild-type strain after 48-h growth in liquid medium (Fig. 3D). These results indicate that NsdC is involved in suppression of conidiation in liquid culture but also has a role in promoting hyphal vegetative growth and concomitant asexual sporulation on solid media (as per Fig. 1A) and that *nsdC* expression is, at least to some degree, dependent on MpkB.

### NsdC participates in organization of the cell wall.

**FIG 4:**
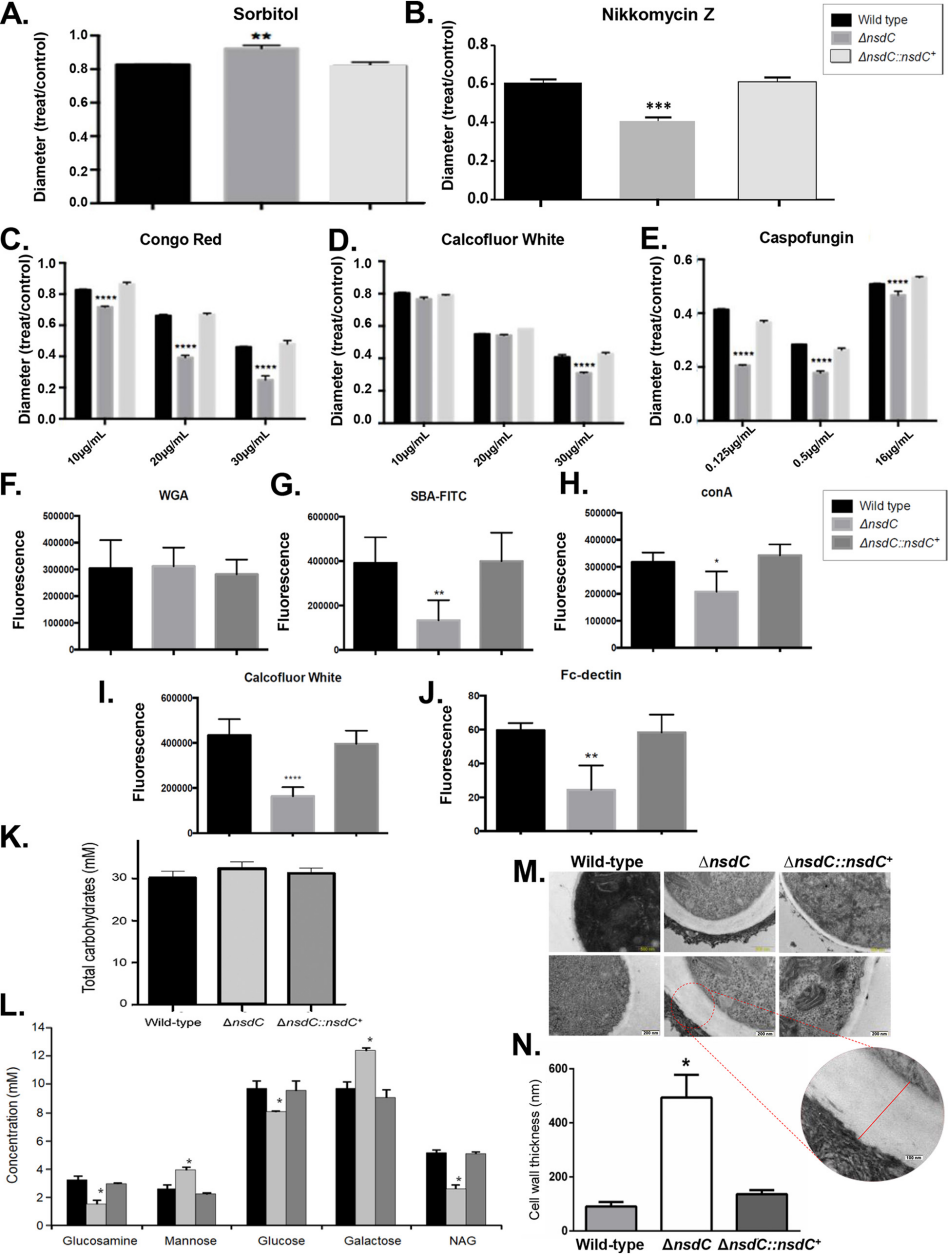
A. fumigatus NsdC affects the cell wall organization and structure. (A to E) The wild-type, Δ*nsdC*, and Δ*nsdC*::*nsdC*^+^ strains were grown for 5 days at 37°C in MM + sorbitol at 1.2 M (A), MM + nikkomycin Z at 100 μM (B), MM + Congo red up to 30 μg/ml (C), MM + calcofluor white up to 30 μg/ml (D), and MM + caspofungin up to 16 μg/ml (E). Error bars represent standard deviations of the average of three independent biological repetitions. Statistical analysis was performed using a one-way ANOVA compared to the wild-type condition (**, *P* < 0.01; ***, *P* < 0.001; ****, *P* < 0.0001). (F to J) Detection of sugar exposed on the cell wall. Conidia were cultured in liquid MM until hyphae were produced, UV killed, and stained with WGA (wheat germ agglutinin)-FITC (recognizing surface-exposed glucosamine [Glc]) (F), SBA (soybean agglutinin)-FITC (preferentially binds to oligosaccharide structures with terminal α- or β-linked *N*-acetylgalactosamine [GalNAc] and, to a lesser extent, galactose residues—important for recognizing galactosaminogalactan [GAG]) (G), ConA (concanavalin A)-FITC (recognizes α-linked mannose) (H), CFW (recognizing chitin) (I), and soluble dectin-1 staining (recognizing β-glucans) (J). (K) Total carbohydrate concentration in the cell walls of the wild-type, Δ*nsdC*, and Δ*nsdC*::*nsdC*^+^ strains. (L) Sugar concentrations in the cell walls as determined by high-performance liquid chromatography (HPLC), in mycelial extracts of the A. fumigatus strains when grown for 16 h in MM at 37°C. Experiments were performed in triplicate, and results are displayed as mean values with standard deviations (one-way ANOVA followed by Tukey’s *post hoc test* [*, *P < *0.05; **, *P* < 0.01; ***, *P* < 0.001; ****, *P* < 0.0001]). (M) Transmission electron microscopy of mycelial sections of A. fumigatus wild-type, Δ*nsdC*, and Δ*nsdC*::*nsdC*^+^ strains following growth for 24 h in MM at 37°C. Inset shows a magnification of the Δ*nsdC* mutant cell wall. (N) The cell wall thickness (nanometers) of 100 sections of different hyphal germlings (average from 4 sections per germling) was measured. Standard deviations present the average from the 100 measurements, and statistical differences were evaluated by using one-way analysis of variance (ANOVA) and Tukey’s *post hoc* test (*, *P < *0.05).

Although 1.2 M sorbitol improved Δ*nsdC* mutant radial growth (Fig. 4A), the mutant strain was more sensitive to the chitin synthase inhibitor nikkomycin Z (Fig. 4B) and other cell wall-damaging agents, including Congo red (CR), calcofluor white (CFW), and caspofungin than were the wild-type and complemented strains (Fig. 4C to E). Increased sensitivity to cell wall stressors suggested the Δ*nsdC* mutant had an altered cell wall composition. Cell wall stains and fluorescently labeled lectins were therefore used to identify differences in the ability to detect (i.e., exposure of) several carbohydrates on the cell wall surface, for the wild-type, Δ*nsdC*, and complemented strains. These included (i) WGA (wheat germ agglutinin)-FITC (fluorescein isothiocyanate), recognizing surface-exposed glucosamine (Glc); (ii) SBA (soybean agglutinin)-FITC, which binds preferentially to oligosaccharide structures with terminal α- or β-linked *N*-acetylgalactosamine (GalNAc) and to galactose residues which are important for recognizing galactosaminogalactan (GAG); (iii) ConA (concanavalin A)-FITC, which recognizes α-linked mannose; (iv) CFW (recognizing chitin); and (iv) soluble dectin-1 staining (recognizing β-glucans). Despite finding no differences in the exposed Glc among all strains (Fig. 4F), the Δ*nsdC* mutant had about a 2- to 3-fold reduction in the exposure of GalNAc, α-linked mannose, chitin, and β-1,3-glucan compared to the wild-type and complemented strains (Fig. 4G to J). All the three strains have about the same amount of total carbohydrates in their cell wall (Fig. 4K); however, the mutant strain displayed lower exposure of the cell wall sugar components glucosamine, glucose, and NAG (*N*-acetylglucosamine) but increased mannose and galactose relative to the wild-type and complemented strains (Fig. 4L). Interestingly, transmission electron microscopy (TEM) experiments showed that the cell wall of the Δ*nsdC* mutant was about 5-fold thicker than those of the wild-type and complemented strains (Fig. 4M and N; see also Table S3 at https://doi.org/10.6084/m9.figshare.12931754.v3). Therefore, NsdC influences fungal cell wall composition organization and structure.

### Chromatin immunoprecipitation of RNA polymerase II coupled to next-generation sequencing (PolII ChIP-Seq) for Δ*nsdC*.

**FIG 5:**
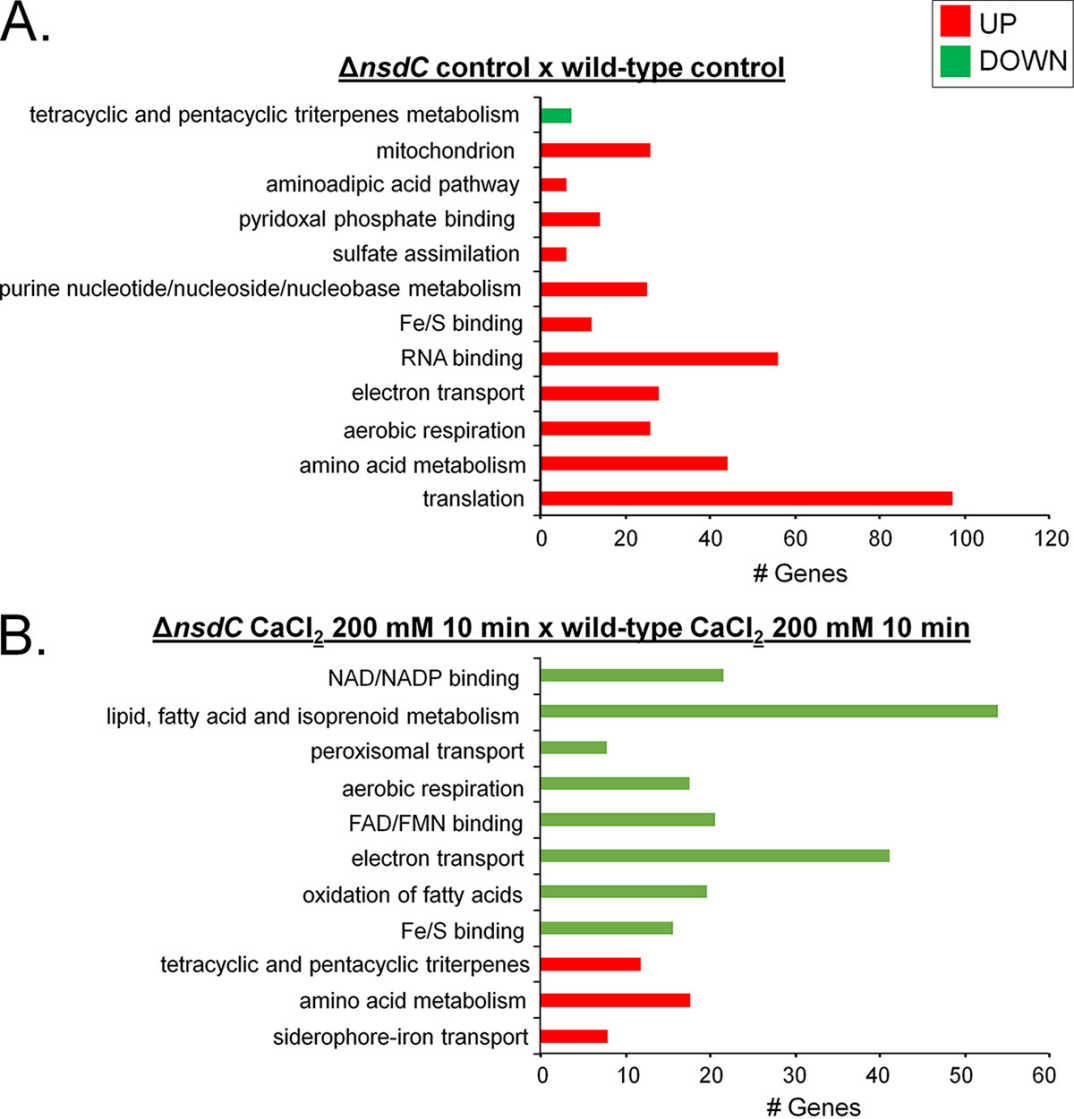
A summary of the categorization terms of overrepresented up- or downregulated (adjusted *P* value < 0.05) genes after PolII ChIP-Seq for the Δ*nsdC* strain relative to the parental control. FunCat (https://elbe.hki-jena.de/fungifun/fungifun.php) enrichment analyses for the Δ*nsdC* control × wild-type control (A) and Δ*nsdC* CaCl_2_ (200 mM, 10 min) × wild-type CaCl_2_ (200 mM, 10 min) (B). FAD/FMN, flavin adenine dinucleotide/flavin mononucleotide.

Considering that NsdC has many different functions besides its involvement in the sexual process, we decided to evaluate the transcriptional impact of the lack of *nsdC* after growth in MM for 16 h in the absence and presence of CaCl_2_. To investigate this, we used PolII ChIP-Seq with a corresponding wild-type strain and the Δ*nsdC* mutant. Considering that RNA PolII recruitment is related to transcriptional activity, a corresponding increase and decrease in RNA PolII occupancy indicate upregulation and downregulation of gene expression, respectively (31, 32). In the Δ*nsdC* strain compared with the wild type, we identified an increase in RNA PolII occupancy of 805 genes and reduction of PolII occupancy of 120 genes (log_2_FC [fold change] ≥ 1.0 and ≤ −1.0; false-discovery rate [FDR] of 0.05; see Table S4 at https://doi.org/10.6084/m9.figshare.12931754.v3). FunCat (https://elbe.hki-jena.de/fungifun/fungifun.php) enrichment analyses of the upregulated genes demonstrated an increased PolII occupancy for those encoding proteins involved in translation, mitochondrion, aerobic respiration, electron transport, and amino acid metabolism (Fig. 5A). FunCat categorization of the downregulated genes allowed us to identify only a single category of genes, in this case encoding proteins involved in tetracyclic and pentacyclic triterpene (cholesterin, steroid, and hopanoid) metabolism (Fig. 5A).

The same method of transcriptional profiling was then used to compare gene expressions of the Δ*nsdC* and wild-type strains when both were exposed to 200 mM CaCl_2_ for 10 min. An increased RNA PolII occupancy was identified for 310 genes together with a reduction of PolII occupancy of 623 genes (log_2_FC ≥ 1.0 and ≤ −1.0; FDR of 0.05; see Table S4 at https://doi.org/10.6084/m9.figshare.12931754.v3). FunCat (https://elbe.hki-jena.de/fungifun/fungifun.php) enrichment analyses demonstrated an increased PolII occupancy of genes encoding siderophore-iron transport and amino acid and tetracyclic and pentacyclic triterpene (cholesterin, steroid, and hopanoid) metabolism (Fig. 5B). However, we have not observed any iron deficiency in the Δ*nsdC* strain radial growth (see Fig. S2 at https://doi.org/10.6084/m9.figshare.12931754.v3). FunCat categorization of the downregulated genes allowed us to identify proteins involved in Fe/S binding; electron transport; aerobic respiration; lipid, fatty acid, and isoprenoid metabolism; peroxisomal transport; amino acid metabolism; mitochondrial transport; and siderophore-iron transport (Fig. 5B).

We identified at least five genes encoding proteins involved in the sexual process as modulated under both transcriptional conditions (absence or presence of CaCl_2_ [Fig. 5A]; also see Table S4 at https://doi.org/10.6084/m9.figshare.12931754.v3): (i) *rosA* (AFUA_4g09710), overexpressed in the presence of CaCl_2_, a repressor of sexual development in A. nidulans (33); (ii) *csnC* (AFUA_2G07340), overexpressed in the absence of CaCl_2_, an A. nidulans ortholog that has a role in cleistothecium development and COP9 signalosome localization (34); (iii) *osaA* (AFUA_3g09640), overexpressed in the presence of CaCl_2_, encoding a protein with a WOPR domain involved in regulation of sexual development in A. nidulans (35); (iv) *nsdD* (AFUA_3g13870), overexpressed both in the absence and in the presence of CaCl_2_, encoding a GATA-type transcriptional activator and required during an early stage of mating (36); and (v) *imeB* (AFUA_2g13140), overexpressed in the presence of CaCl_2_, a serine/threonine protein kinase involved in the inhibition of sexual development in A. nidulans (37).

**FIG 6:**
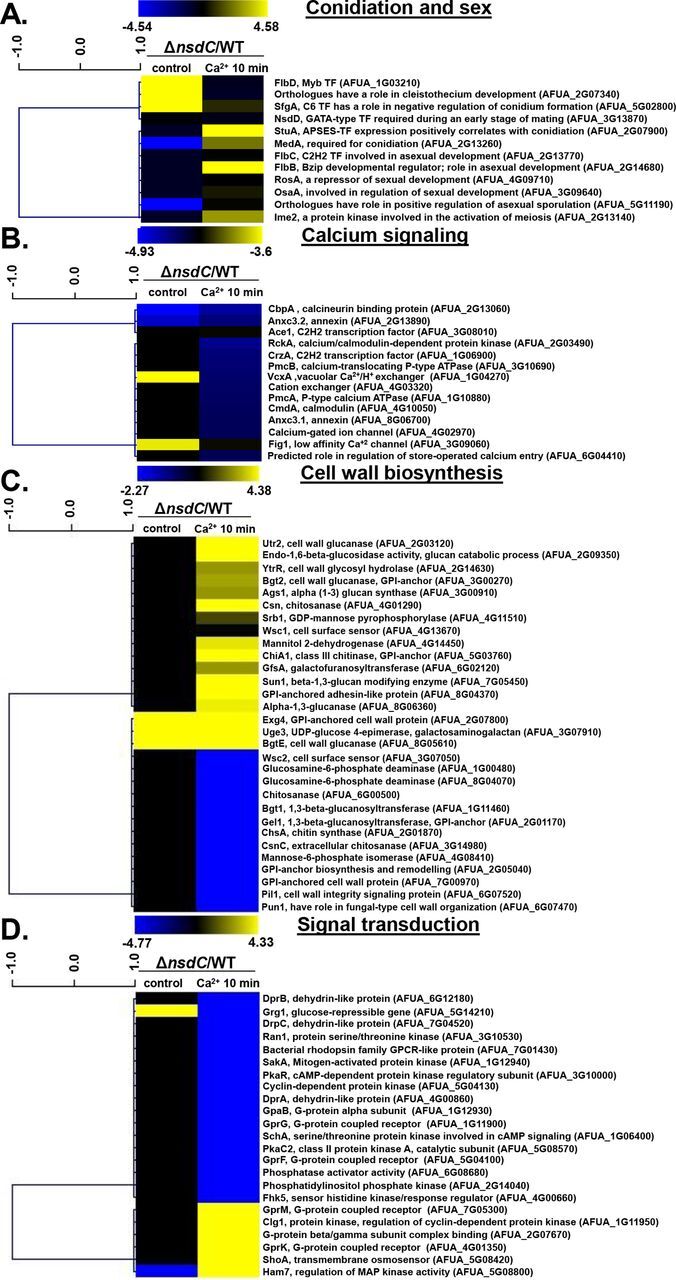

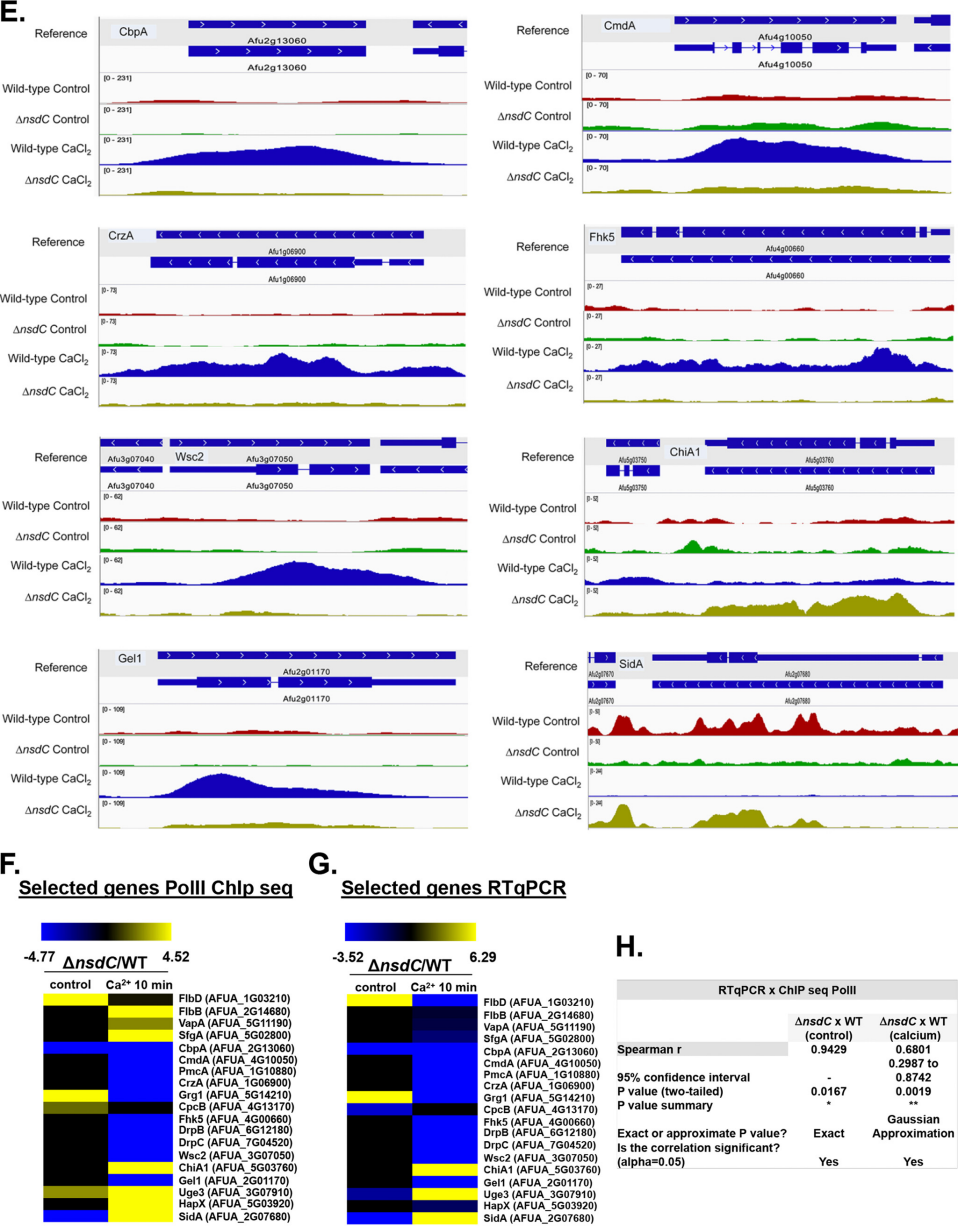
Independent validation of selected genes identified as modulated in PolII ChIP by RT-qPCR. (A to D) The results show the PolII occupancy for selected annotated genes encoding proteins putatively involved in conidiation and sex (A), calcium signaling (B), cell wall biosynthesis (C), and signal transduction (D). The Δ*nsdC* strain is shown as the deletion strain versus the equivalent wild-type (WT) strain for control and 10-min time point (the mutant values have been normalized to the basal level of expression of each gene before stress, i.e., expression ratios are being compared: the Δ*nsdC* control divided by the wild-type control and the Δ*nsdC* mutant 10 min versus time zero divided by the wild-type 10 min versus time zero). Hierarchical clustering was performed in MeV (http://mev.tm4.org/), using Spearman correlation with complete linkage clustering. (E) PolII ChIP-Seq Integrative Genomics Viewer (IGV; http://software.broadinstitute.org/software/igv/download) screenshot for selected genes from panels F and G. (F) Heat map for the PolII occupancy of 19 selected genes involved in conidiation and sex, calcium signaling, cell wall biosynthesis, and signal transduction. (G) Heat map of RT-qPCR for the same 19 selected genes from panel F. The wild-type and Δ*nsdC* mutant strains were grown for 20 h at 37°C and transferred to 200 mM CaCl_2_ for 0 and 10 min. Gene expression was normalized using *cofA* (Afu5g10570). Standard deviations present the average of three independent biological repetitions (each with 2 technical repetitions). (H) The expression of these 19 genes as measured by RT-qPCR showed a high level of correlation with the PolII ChIP-Seq data (Spearman correlation from 0.6801 to 0.9429).

There are several genes encoding proteins involved in the asexual conidiation process that are overexpressed in the absence of CaCl_2_ (Fig. 6A; also see Table S4 at https://doi.org/10.6084/m9.figshare.12931754.v3), such as *flbD* (AFUA_1G03210, a Myb family transcription factor whose A. nidulans ortholog plays a role in conidiophore development [38]) and SfgA (AFUA_5G02800, a C6 transcription factor that has a role in negative regulation of conidium formation [39]), and in the presence of CaCl_2_, such as *flbB* (AFUA_2g16060, a Bzip developmental regulator involved in A. nidulans asexual development [40]), *flbC* (AFUA_2g13770, a C_2_H_2_ finger domain protein involved in asexual development in A. nidulans [40]), and *vapA* (AFUA_5g11190, a component of the plasma membrane-associated VapA-VipC-VapB methyltransferase complex that controls A. nidulans differentiation [41]).

Taken together, these results indicate that NsdC is important for amino acid, iron, and lipid metabolism as well as conidiation, impacting several biological responses under basal conditions and upon exposure to calcium stress.

### Independent validation of NsdC influence on transcriptional regulation of calcium response.

RT-qPCR experiments validated the PolII ChIP-Seq results for the majority of the 19 selected genes from conidiation and sex, calcium signaling, cell wall, and signal transduction responses (Fig. 6A to G; see also Table S4 and Fig. S5 at https://doi.org/10.6084/m9.figshare.12931754.v3). The *cofA* (Afu5g10570) gene, which encodes the actin-binding protein cofilin, was used as a normalizer due to its consistent expression in all strains during calcium stress (30). The expression of these 19 genes showed a high level of correlation with the PolII ChIP-Seq data (Spearman correlation from 0.6801 to 0.9429 [Fig. 6H]). These results validate that NsdC has functions in modulating the response to calcium stress, affecting directly or indirectly the expression of genes involved in conidiation, sex, signal transduction, and cell wall biosynthesis and/or remodeling.

### The A. fumigatus Δ*nsdC* mutant has attenuated virulence in immunodeficient mice.

**FIG 7:**
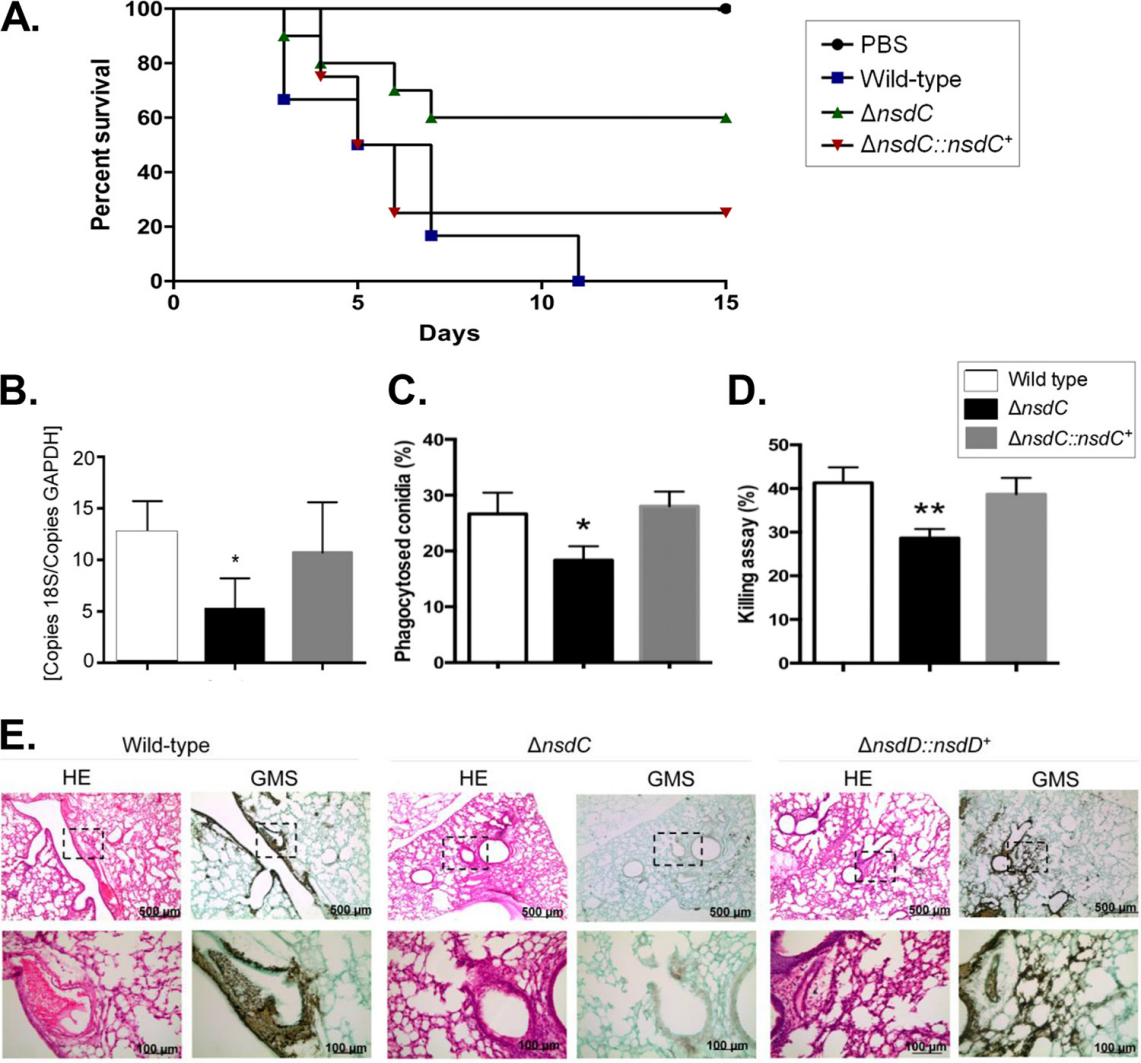
The A. fumigatus Δ*nsdC* strain has attenuated virulence in neutropenic mice. (A) Comparative analysis of wild-type, Δ*nsdC*, and Δ*nsdC*::*nsdC*^+^ strains in a neutropenic murine model of pulmonary aspergillosis. Mice in groups of 10 per strain were inoculated intranasally with a 20-μl suspension of conidia at a dose of 10^5^. PBS, phosphate-buffered saline. The statistical significance of comparative survival values was calculated with the Prism statistical analysis package by using log rank (Mantel-Cox) and Gehan-Brestow-Wilcoxon tests. (B) Fungal burden was determined 48 h postinfection by real-time qPCR based on the 18S rRNA gene of A. fumigatus and an intronic region of the mouse GAPDH gene. Fungal and mouse DNA quantities were obtained from the *C_T_* values from an appropriate standard curve. Fungal burden was determined through the ratio between nanograms of fungal DNA and milligrams of mouse DNA. The results are the means (± standard deviations) from five lungs for each treatment. Statistical analysis was performed by using the *t* test (*, *P* < 0.05). (C and D) Fungal killing capacity of mouse bone marrow-derived macrophages (BMDMs) assessed as CFU of surviving A. fumigatus strains (wild type, Δ*nsdC*, or Δ*nscD*::*nscD*^+^ mutant) after 4 h of infection. The percent killing was calculated from CFU remaining compared with control samples without macrophages. The results are the means (± standard deviation) of three independent biological repetitions. Statistical analysis was performed using a one-way ANOVA compared to the wild-type condition (*, *P* < 0.05; **, *P* < 0.01). (E) Histopathology of mice infected with A. fumigatus wild-type or Δ*nsdC* or Δ*nscD*::*nscD*^+^ mutant strain. GMS (Grocott’s methenamine silver) and HE (hematoxylin and eosin) staining of lung sections representative of infections. The boxed area from the top row is magnified in the bottom row. Bars, 100 and 500 μm.

In the leukopenic BALB/c murine model of invasive pulmonary aspergillosis, wild-type and Δ*nsdC*::*nsdC*^+^ exposure resulted in 100 and 80% mortality at 11 and 15 days postinoculation, respectively (Fig. 7A). In contrast, the Δ*nsdC* mutant caused a reduced mortality of only 40% at 15 days postinoculation, which was statistically different from the wild-type and Δ*nsdC*::*nsdC*^+^ strains (Mantel-Cox and Gehan-Brestow-Wilcoxon tests; *P* values < 0.05 [Fig. 7A]). Additionally, fungal burden in lungs of mice was measured by qPCR and revealed a lower presence of Δ*nsdC* DNA than of that of the wild-type and complemented Δ*nsdC*::*nsdC^+^* strains (Fig. 7B). These data strongly indicated that the lack of NsdC in A. fumigatus caused a significant reduction in virulence in immunodeficient mice and also indicated that the Δ*nsdC* strain might be more sensitive to macrophage killing.

Histopathological examination revealed that at 72 h postinfection mouse lungs infiltrated with the Δ*nsdC* strain showed no sign of fungal burden. In contrast, mice infected with the wild-type or the Δ*nsdC*::*nsdC^+^* strain contained multiple foci of invasive hyphal growth, which penetrated the pulmonary epithelium in major airways (Fig. 7E). These data strongly indicated that the lack of NsdC in A. fumigatus caused a significant reduction in virulence in immunodeficient mice and also indicated that the Δ*nsdC* strain might be more sensitive to macrophage killing.

Since loss of the *nsdC* gene produced alterations in cell wall composition (Fig. 4), we hypothesized that it could influence the immune host response and virulence. Macrophages contribute to innate immunity, fungal clearance, and the generation of a proinflammatory response during A. fumigatus exposure to the microbe (42). Thus, the capacity of bone marrow-derived macrophages (BMDMs) to phagocytose and kill the wild-type, Δ*nsdC*, and Δ*nsdC*::*nsdC*^+^ conidia was assessed. The *nsdC* mutant conidia presented lower killing rates by BMDMs than did the wild-type and *nsdC*::*nsdC^+^* conidia (Fig. 7C and D). These results indicate that the *nsdC* mutant was less susceptible to macrophage killing.

**FIG 8:**
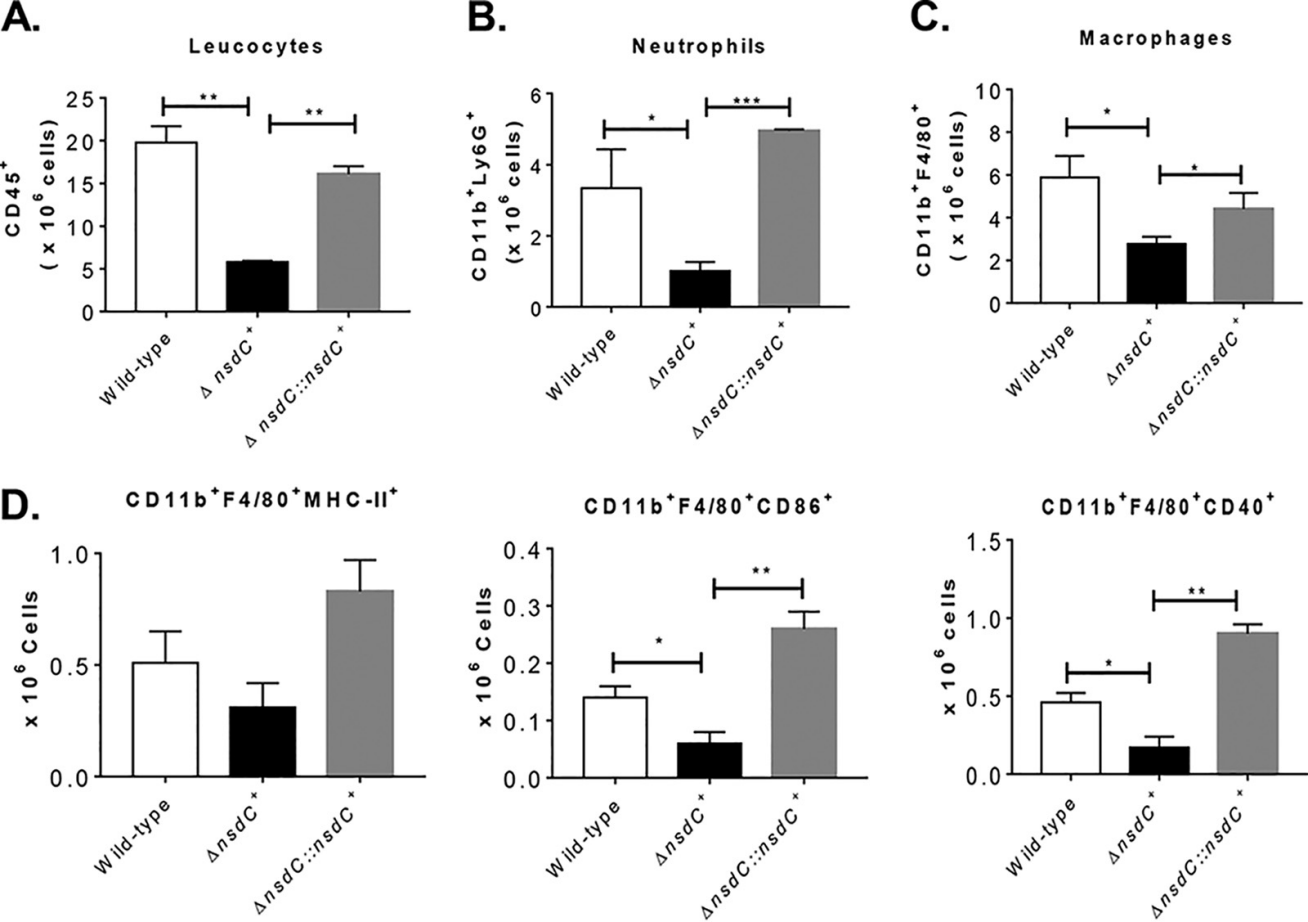
Δ*nsdC* determines impaired leukocyte influx to the lungs of immunocompetent C57BL/6 mice. Mice were inoculated with 5 × 10^7^ conidia of A. fumigatus strains by i.t. route, and cell phenotypes were determined after 72 h of infection. Lungs from mouse groups (*n* = 3) were excised and digested enzymatically. Cell suspensions were obtained and stained as described in Materials and Methods. The stained cells were analyzed immediately on a FACSLyrics flow cytometer with gating of granulocytes, as judged from forward scatter (FSC) and side scatter (SSC). The number of total CD45^+^ leukocytes (A), CD11b^+^ F4/80^−^ Ly6G^+^ neutrophils (B), CD11b^+^ F4/80^+^ macrophages (C), and inflammatory macrophages (CD11b^+^ F4/80^+^ MHC-II^+^, CD11b^+^ F4/80^+^ CD86^+^, and CD11b^+^ F4/80^+^ CD40^+^, respectively) (D) was assessed. One hundred thousand cells were counted, and the data are expressed as number of positive cells. Data are expressed as means ± standard errors (SE) of the mean and are representative of two independent experiments (*, *P < *0.05, and **, *P* < 0.01).

In addition, experiments with immunocompetent C57BL/6 mice were performed to assess the ability of the Δ*nsdC* strain to generate an inflammatory response. The immunocompetent mice were inoculated with 5 × 10^7^ conidia of wild-type, Δ*nsdC*, and Δ*nsdC*::*nsdC^+^* strains by the intratracheal (i.t.) route. After 72 h of inoculation, the lung-infiltrating leukocytes were obtained and analyzed for the expression of surface molecules of leukocytes by flow cytometry. As shown in Fig. 8A to D and also in Fig. S3 at https://doi.org/10.6084/m9.figshare.12931754.v3, lower numbers of total leukocytes (CD45^+^ cells), neutrophils, and macrophages were found in the lungs of mice exposed to Δ*nsdC* mutant strains compared with wild-type and Δ*nsdC*::*nsdC^+^* conidium inoculation. Similarly, fewer numbers of inflammatory macrophage cells (CD11b^+^ F4/80^+^ major histocompatibility complex class II [MHC-II^+^], CD11b^+^ F4/80^+^ CD86^+^, and CD11b^+^ F4/80^+^ CD40^+^, respectively) were detected in the lungs of mice inoculated with Δ*nsdC* mutant strains compared with wild-type and Δ*nsdC*::*nsdC* controls. These data strongly indicate that the lack of NsdC in A. fumigatus caused a significant reduction in virulence in immunodeficient and immunocompetent mice and also indicated that the Δ*nsdC* strain was more sensitive to macrophage killing.

## DISCUSSION

A family of 20 Nsd mutants were first identified in A. nidulans and named based on the fact that they failed to produce cleistothecia, i.e., were never in sexual development (43, 44). Of these mutants, only the NsdC and NsdD mutants have so far been characterized (29, 44, 45). Partly due to the nomenclature used, both the *nsdC* and *nsdD* genes are known primarily for their essential role in sexual reproduction (27). However, results from the present study, combined with reevaluation of previous studies of *nsdC* gene function, reveal that the NsdC transcription factor in particular has much more broad biological functions beyond simply promotion of sexual reproduction, as will now be described.

An indication of one of the additional functions of NsdC came recently from a study aiming to identify new transcription factors involved in the calcium stress response in A. fumigatus (30). Large-scale phenotypic screenings have been widely used to identify new components of signaling pathways and to study drug resistance mechanisms (46–49). A library of null-mutant transcription factors was challenged with high concentrations of CaCl_2_, and this resulted in the identification of an A. fumigatus Δ*nsdC* mutant with increased sensitivity to CaCl_2_ relative to the wild-type parental strain, suggesting a role for NsdC in calcium response. Additional work in the present study demonstrated that complementation with a functional *nsdC* gene restored the wild-type phenotype. Further phenotyping assays then showed that loss of *nsdC* led to cyclosporine (a calcineurin-inhibitory drug) resistance. These combined results indicate a first additional important role for NsdC in mediating calcium tolerance. Whether this involves a direct or indirect link to the central calcium-calcineurin-CrzA pathway requires further investigation. Additional experiments are necessary to characterize the interactions among these different TFs and calcineurin. Interestingly, transcriptional profiling using PolII ChIP-Seq showed at least 933 changes in gene expression between the Δ*nsdC* mutant and the wild type when exposed to calcium stress, indicating a possible wide spectrum of activity for NsdC in calcium response. However, most of these changes are in increased gene expression (805 changes), suggesting that NsdC could act as a repressor.

Given that *nsdC* was first identified in A. nidulans due to a total lack of sexual development (including failure to produce Hülle cells as well as cleistothecia) even under conditions which favor fruiting body formation by single isolates (29), we then wished to determine whether NsdC had a similar role in sexual development of A. fumigatus. Despite sexual development being demonstrated in A. fumigatus under laboratory conditions (22), most advances in understanding the regulation of the sexual cycle in *Aspergillus* species have come from studies using the model A. nidulans (27, 44, 50). However, notable differences in the breeding systems of the species (A. fumigatus is heterothallic whereas A. nidulans is homothallic) suggested that findings from A. nidulans might not necessarily be applicable to A. fumigatus. Therefore, both *MAT1-1* and *MAT1-2* Δ*nsdC* mutant strains of A. fumigatus were constructed in a high-fertility genetic background to assess the contribution of NsdC in sexual reproduction in this species. It was found that, provided one of the *MAT1-1* or *MAT1-2* mating partners contained a functional copy of *nsdC*, the sexual cycle could be completed, leading to cleistothecium and ascospore production, i.e., the absence of NsdC in one mating partner could be complemented by the presence of NsdC produced by the other partner. Indeed, in the original work of Kim et al. (29) it was found that despite the failure to undergo sexual development under selfing conditions, an *nsdC* deletion mutant of A. nidulans was nevertheless still able to outcross to a compatible auxotrophic strain of A. nidulans with a functional copy of *nsdC*, mirroring our findings with A. fumigatus. The exact mechanism of complementation is unclear, but it can be speculated that the partner with the functional copy of *nsdC* might act as the maternal partner, forming ascogonial coils (and later maternal tissues) that could be fertilized by the male Δ*nsdC* partner, given that *nsdC* is thought to act at a very early stage of sexual development (27, 29). Alternatively, the partner with the functional copy of *nsdC* might allow hyphal fusions necessary for sex to occur, with this ability missing from Δ*nsdC* strains. It has proved difficult to determine the early morphological stages of sexual development in A. fumigatus (K. M. Lord, N. D. Read, and P. S. Dyer, unpublished results), although putative ascogonial coils have been described in A. nidulans (51). However, if both the *MAT1-1* and *MAT1-2* mating partners of A. fumigatus were of the Δ*nsdC* genotype, then sexual fertility was totally abolished. This was a significant result, as it demonstrated that NsdC has a second, essential positive regulatory role for sexual development in A. fumigatus, as observed for its counterpart in A. nidulans (29). NsdC has also been shown to have a role in sexual development in A. flavus, where its deletion led to inhibition of formation of sclerotia, these structures being an important first stage in the sexual cycle (27, 52).

These findings also provide a key precedent when evaluating the function of genes in sexual development in the heterothallic A. fumigatus, in that it will be important to disrupt the gene(s) under study in both mating partners to conclusively evaluate gene function. Indeed, it is noted that the function of genes in sexual development is generally performed with homothallic species such as A. nidulans, Sordaria macrospora, and Fusarium graminearum (27, 53, 54) as this removes the time-consuming need to delete a gene(s) in both partners, which is the case for heterothallic species. However, there may be exceptions such as studies of functionality of mating-type genes, as distinct *MAT1-1* and *MAT1-2* idiomorph genes are found in the different mating partners (20, 25, 55). Also, in contrast to the current results for *nsdC*, the loss of the *nsdD* gene (another transcription factor involved in sexual reproduction in the aspergilli) in one mating partner alone was enough to suppress fruit body formation in A. fumigatus (25).

In the original study of *nsdC* function in A. nidulans, Δ*nsdC* mutants were also found to be characterized by retarded vegetative growth compared to the wild type, with an approximately 30% reduction in growth rate (29). A very similar result was found in the present study, with the A. fumigatus Δ*nsdC* strains in both the CEA17 and supermater genetic backgrounds showing a reduction in vegetative growth rate on solid media. However, this growth difference is observed only in minimal medium and not in complete medium, suggesting the Δ*nsdC* mutant has metabolic deficiencies that are supplemented by the complete medium chemical composition. Taken together, these results confirm a third important role for NsdC in the promotion of vegetative growth. This conclusion is supported in part by the gene expression studies from the present study, involving PolII ChIP-Seq, which showed that NsdC is associated with the regulation of several biological processes under basal growth conditions. It is speculated that the upregulation of some biological functions in the Δ*nsdC* mutant was perhaps to counterbalance the loss of NsdC. Interestingly, biosynthesis of different aspects of secondary metabolism was up- and downregulated in the Δ*nsdC* mutant, implying a regulatory function of NsdC in secondary metabolite production as has been observed in Aspergillus flavus (52).

In the original study of *nsdC* function in A. nidulans, Δ*nsdC* mutants were furthermore found to be characterized by “hyperactive” asexual sporulation, with earlier-than-normal development of conidia, and conidiation even in liquid media, indicating that NsdC negatively regulates asexual reproduction (29). A very similar result was found in the present study with A. fumigatus, with microscopic examination revealing the production of conidiophores and conidia in the Δ*nsdC* mutant when grown in liquid MM, conditions under which the parental wild type failed to produce conidia. This activity of NsdC appears to be explained in part as a result of repression of *brlA* expression. BrlA is a key regulator of asexual sporulation due to its activity in controlling the initiation of conidiophore development (6, 9, 11, 56). It was found that *brlA* mRNA accumulated in the Δ*nsdC* strain after 24 h of growth in liquid MM, unlike the parental strain. Higher *brlA* expression has also been observed in an Δ*nsdC* mutant of A. flavus (which also exhibited altered conidiophore morphology), and *brlA* transcripts were found to be expressed earlier (but in similar quantities) in an A. nidulans Δ*nsdC* mutant (29, 52). Transcriptional profiling also indicated that several genes important for conidiation, such as *flbB*, *flbC*, and *flbD*, have increased expression in the Δ*nsdC* mutant. Meanwhile, our group and others have previously described a role of the MAP kinase MpkB in the negative regulation of asexual development in both A. fumigatus and A. nidulans, whereby Δ*mpkB* mutants produced conidia in submerged cultures with a constant accumulation of *brlA* mRNA (18, 57). In the present study, we observed an accumulation of *nsdC* mRNA in the Δ*mpkB* strain of A. fumigatus, indicating negative control of *nsdC* expression by MpkB. Further investigation is required in order to establish if MpkB also influences sexual development in A. fumigatus, as already described for A. nidulans (28). Taken together, these results confirm a fourth important role for NsdC in the negative regulation of asexual reproduction by transcriptional repression of *brlA*, subject to control by MpkB.

Cell wall composition and structure vary substantially with cell cycle progression and in response to environmental changes (58). Very little is known about A. fumigatus TFs regulating cell wall biosynthesis and/or remodeling. A. fumigatus TFs such as RlmA and calcium-calcineurin-dependent TFs such as CrzA and ZipD have been characterized in more detail as regulating several genes involved in the cell wall metabolism (30, 49, 59–66). In the present study, deletion of the *nsdC* gene led to greater sensitivity to cell wall-damaging agents, a reorganization of cell wall structure, decreased and increased cell wall sugar content, and increased cell wall width. The A. fumigatus cell wall is composed of more than 90% polysaccharides, with the core skeleton composed by a branched β-1,3-glucan linked to chitin, galactomannan, and β-1,3-β-1,4-glucans; α-1,3-glucan and mannans act as a cement, filling the pores between fibrillar polysaccharides (67). We have observed no differences in total carbohydrates isolated from the cell walls of the wild-type, Δ*nsdC*, and complemented strains. However, there are increased concentrations of mannose and galactose and decreased concentrations of glucosamine, glucose, and NAG present in the Δ*nsdC* cell walls compared to the wild-type and complemented strains. It proved difficult to establish a direct relationship between the organization and composition of the fungal cell wall that could directly explain the observed increased thickness of Δ*nsdC* cell walls. It is possible that the combination of the increased and decreased sugar concentrations could impact the Δ*nsdC* cell wall organization, increasing the cell wall width. Transcriptional profiling also showed that several genes involved in the cell wall biosynthesis and remodeling were dysregulated in the Δ*nsdC* mutant. Cell wall thickening has also been linked to defects in sphingolipid metabolism in A. nidulans and yeasts (68), but alteration of cell membrane components was not assessed in this work. Interestingly, transcriptional profiling found up- and downregulated genes involved in lipid, fatty acid, and isoprenoid metabolism, which could help to explain this striking phenotype. In contrast, loss of *nsdD* in A. fumigatus conferred resistance toward certain cell wall stressors (25). Taken as a whole, these results demonstrate a fifth important role for NsdC in response to cell wall stress and correct cell wall organization. More studies are necessary to understand how NsdC interacts with RlmA, CrzA, and ZipD; is regulated by calcineurin; and populates the promoter regions of the genes encoding proteins important for the cell wall integrity pathway.

Cell wall organization impacts directly virulence and host immune recognition in A. fumigatus (69). Accordingly, leukopenic mice inoculated with Δ*nsdC* conidia showed a reduction in mortality, fungal burden, and inflammation compared to inoculation with the wild-type parental strain, possibly also linked to the lower growth rate of the Δ*nsdC* strain. Despite the reduced virulence, Δ*nsdC* conidia were less well recognized by macrophages than wild-type conidia, probably because of the increased thickness of the cell wall in this mutant which possibly masks β-1,3-glucan (which is already reduced in the mutant) and other cell wall components responsible for triggering immune response in the host (70). As a result, Δ*nsdC* conidia were also less efficiently eliminated. This impaired interaction between macrophages and Δ*nsdC* conidia likely resulted in fewer inflammatory cytokines and consequently a diminished influx of leukocytes to the site of inoculation as further confirmed by results from the immunocompetent model of pulmonary acute aspergillosis included in the present study. Corroborating the lower phagocytosis index, the killing rates may be influenced not just because the conidia were not killed but also because the conidium ingestion was affected. However, a compromised phagocytosis associated with an efficient macrophage activity would affect the recruitment of immune cells as shown in Fig. 6A. The impaired interaction between alveolar macrophages and the *nsdC* mutant strain likely resulted in reduced inflammatory signals and consequently less influx of leucocytes to the site of infection as we showed in Fig. 6. In addition, the diminished expression of activation markers (Fig. 6D) clearly showed that mechanisms of macrophage activation were impaired against the *nsdC* mutant strain. We believe that the cell wall organization impacts directly virulence and host immune recognition by innate immune cells during A. fumigatus infection. These findings were previously described and revised by reference 69. These results demonstrate an extra sixth role for NsdC in virulence and host immune recognition.

In summary, in addition to its known essential roles in sexual reproduction and control of growth rate and asexual reproduction, we have shown in the present study of A. fumigatus that the NsdC transcription factor has additional previously unrecognized biological functions including calcium tolerance, cell wall stress response, and correct cell wall organization and functions in virulence and host immune recognition. Indeed, the fact that NsdC is needed for correct vegetative growth and that gene deletion resulted in changes in expression of over 620 genes under basal growth conditions suggests that promotion of sexual reproduction is perhaps not the principal role of NsdC (despite the gene epithet) and that instead this transcription factor mediates the activity of a wide variety of key signaling and metabolic pathways. In contrast, deletion of many other genes in A. nidulans with a principal role in sexual reproduction has little impact on vegetative growth (27). In addition, whereas overexpression of the key sex-related genes *veA* and *nsdD* resulted in induction of sex in submerged cultures of A. nidulans, no such effect was seen with overexpression of *nsdC*, again consistent with a primary role other than sexual reproduction (29). The multifunctionality of NsdC has been proposed to be linked to the transcription of two different mRNA forms from the *nsdC* gene in A. nidulans, with a 3.0-kb form staying relatively constant during the life cycle whereas a shorter, 2.6-kb form accumulated differentially especially during sterigma and sexual development (29). Further research is now warranted into how gene expression is controlled by the NsdC transcription factor.

## MATERIALS AND METHODS

### Strains and media.

All strains used in this study are listed in either Table S1 or Table S5 at https://doi.org/10.6084/m9.figshare.12931754.v3. Strains for genetic manipulation were grown at 37°C in either complete medium (YG: 2% [wt/vol] glucose, 0.5% [wt/vol] yeast extract, trace elements) or minimal medium (MM: 1% [wt/vol] glucose, original high-nitrate salts, trace elements, pH 6.5). Solid YG and MM were the same as described above except that 2% (wt/vol) agar was added. Trace elements, vitamins, and nitrate salts compositions were as described previously (71). When required, MM was supplemented at stated concentrations with calcium chloride (CaCl_2_), cyclosporine, sorbitol, nikkomycin Z, Congo red (CR), calcofluor white (CFW), and caspofungin. Strains for sexual reproductive purposes were grown at 28°C on *Aspergillus* complete medium for production of conidia for mating inoculum (20). For characterization of phenotype, plates were inoculated with 10^4^ spores per strain and left to grow for 120 h at 37°C.

### Construction of A. fumigatus mutants for Δ*nsdC* growth assays and NsdC-GFP localization.

To generate the NsdC-GFP::pyrG fusion fragment, a 3-kb portion of DNA consisting of the *nsdC* open reading frame (ORF) and 5′ untranslated region (UTR), along with a 1-kb segment of DNA consisting of the 3′ UTR flanking region, was amplified with primers nsdC pRS426 5fw/nsdC orf LINKER GFP rv and nsdC 3utr pyrG 3fw/nsdC pRS426 3rv, respectively, from CEA17 genomic DNA (gDNA). The 3.3-kb linker-GFP-trpC-pyrG fusion was amplified with primers OZG916 and OZG964 from the pOB435 plasmid. The cassette was generated by transforming each fragment along with the plasmid pRS426 cut with BamHI/EcoRI into the Saccharomyces cerevisiae strain. This cassette was then transformed into the CEA17 strain, and verification of NsdC tagging was confirmed via PCR. Figures S4 and S5 at https://doi.org/10.6084/m9.figshare.12931754.v3 show the confirmatory PCR and Southern blots for the deletion, complementation, and GFP fusion strains. All primers used above are described in Table S6 at https://doi.org/10.6084/m9.figshare.12931754.v3.

### Microscopy.

Conidia from the NsdC-GFP strain were cultivated on coverslips in 4 ml of MM for 16 h at 30°C before treatment with 200 mM CaCl_2_ for 15 min, 0.25 μg/ml cyclosporine for 30 min, or 0.25 μg/ml cyclosporine for 30 min and 200 mM CaCl_2_ for 15 min. For colocalization experiments, germlings were costained with Hoechst stain (12 μg/ml) (Life Technologies, Inc.) for 3 min. Coverslips were inspected on an Observer Z1 fluorescence microscope (Carl Zeiss) using the 100× objective oil immersion lens (for GFP, filter set 38 HE, excitation wavelength of 450 nm to 490 nm, and emission wavelength of 525 nm to 550 nm; for Hoechst stain, filter set 49 HE, excitation wavelength of 365 nm, and emission wavelength of 420 nm to 470 nm). Differential interference contrast (DIC) images and fluorescent images were captured with an AxioCam camera (Carl Zeiss, Inc.) and processed using the AxioVision software (version 4.8).

### Conidium count.

Freshly harvested conidia (1 × 10^4^) of the wild-type and Δ*nsdC* and Δ*nsdC*::*nsdC*^+^ mutant strains were inoculated onto solid MM at 37°C for 5 days. After this period of growth, four circular sections of the same size were taken from each plate (approximately 1 cm in diameter each) and placed in a Falcon tube containing 10 ml of a 0.01% Tween solution. Conidia were counted after intensive vortexing using a Neubauer chamber. The assay was performed in triplicate.

### Generation of Δ*nsdC* mutants in a high-fertility *MAT1-1* background.

The highly fertile A. fumigatus *MAT1-1* strain 47-51 (synonym AfIR974 [22]) was used as a recipient strain to generate an Δ*nsdC* mutant. The *nsdC* deletion construct was amplified by Phusion polymerase (Thermo) from plasmid pRS426 with primers Af_nsdCSma_up_f and Af_nsdCSma_do_r (see Table S6 at https://doi.org/10.6084/m9.figshare.12931754.v3), resulting in a PCR product with flanking SmaI restriction sites. The fragment was cloned into the pJET1.2 PCR cloning vector (Thermo) and amplified in Escherichia coli DH5α cells. Plasmid DNA was isolated by using the NucleoSpin plasmid isolation kit (Macherey-Nagel) and restricted with SmaI (FastDigest; Thermo) to release the *nsdC* deletion cassette from the plasmid backbone. The 4,345-bp deletion construct, including the pyrithiamine resistance cassette *ptrA* flanked by the upstream and downstream noncoding regions of the *nsdC* gene, was purified from a 1% agarose gel using the GeneJET gel extraction kit (Thermo), and about 2.5 μg of DNA was used for a polyethylene glycol (PEG)-mediated transformation of A. fumigatus strain 47-51 as described previously (72), with the exception that a mixture of 1.2 g VinoTaste Pro (Novozymes), 0.1 g lysing enzymes from Trichoderma harzianum (Sigma), and 0.1 g Yatalase (TaKaRa/Clontech) was used for the generation of protoplasts. Transformants were selected by the presence of 0.1 μg/ml pyrithiamine (Sigma) in the regeneration medium. Transformants were subsequently analyzed for gene deletion by Southern blot analysis. A digoxigenin (DIG)-labeled probe against the *nsdC* upstream region was amplified by *Taq* polymerase (New England Biolabs) with primers Af_nsdCSma_up_f and nsdCAf_up_r (see Table S6 at https://doi.org/10.6084/m9.figshare.12931754.v3) and a nucleotide mix containing DIG-11-dUTP. Genomic DNA of wild type and transformants was restricted with EcoRI, separated on a 0.8% agarose gel, blotted on a nylon membrane, and hybridized with the digoxigenin-labeled probe. Bands were visualized by using antidigoxigenin Fab fragments linked to alkaline phosphatase and developed by using the chemiluminescent substrate CDP-Star (Sigma). Transformants showing a single band and a shift of the wild-type signal from 5.3 kb down to 2 kb were used in subsequent experiments.

### Generation of Δ*nsdC* mutants with a *MAT1-2* background.

To test the fertility of the Δ*nsdC* mutants in the *MAT1-1* background of the high-fertility strain 47-51 and to generate *nsdC* mutants with a *MAT1-2* background, sexual crosses were set up with high- to medium-fertility *MAT1-2* strains of A. fumigatus, namely, strains 47-55 (synonym AfIR964 [22]) and 47-107 (S. S. Swilaiman, G. Szakacs, and P. S. Dyer, unpublished data) (see Tables S1 and S2 at https://doi.org/10.6084/m9.figshare.12931754.v3). Crosses were set up on oatmeal agar at 30°C as described by Ashton and Dyer (24) with four replicate 9-cm petri plates per cross, together with control crosses with the 47-51 parent. After 3 to 4 months of incubation, total numbers of cleistothecia per plate were counted using a dissecting microscope and “hoovering” conidia from plates to ensure that any cleistothecia were visible (23, 24). Cleistothecia were collected, and ascospores were released into 100 μl sterile 0.05% Tween 80. Ascospores were then heated for 1 h at 70°C to inactivate remaining conidia and vegetative hyphae and to simultaneously induce germination of ascospores (22, 24). Ascospores were plated on *Aspergillus* minimal medium with 50 mM glucose as a carbon source and 10 mM glutamine as a nitrogen source (73) in the presence or absence of 0.1 μg/ml of pyrithiamine, given that pyrithiamine resistance was indicative of deletion of the *nsdC* gene. The mating type of individual colonies was subsequently determined by multiplex PCR as previously described (20).

### Fertility analyses of Δ*nsdC* mutants with either *MAT1-1* or *MAT1-2* mating-type background.

Sexual offspring of *MAT1-1* or *MAT1-2* mating type which were of the Δ*nsdC* genotype were analyzed for sexual fertility in crossing experiments with either wild type or Δ*nsdC* mutants of the opposite mating type. This included the use of an additional high-fertility *MAT1-1* isolate, 47-267 (see Table S1 at https://doi.org/10.6084/m9.figshare.12931754.v3), for testing of the fertility of *MAT1-2* isolates. Four replicate 9-cm petri plates were set up for each cross. The level of fertility was evaluated by counting total numbers of cleistothecia per crossing plate as described above. Resultant data were analyzed using Prism 8.0 by one-way analysis of variance (ANOVA) and nested one-way ANOVA as appropriate.

### RNA extraction and gene expression analysis.

Strains were grown from 1 × 10^7^ conidia in MM for 24 h (wild-type, Δ*nsdC*, Δ*nsdC*::*nsdC*^+^, and Δ*mpkB* strains) or 48 h (wild-type and Δ*mpkB* strains) at 37°C. Mycelia were ground to a fine powder in liquid N_2_, and total RNA was extracted with TRIzol reagent (Thermo Scientific) according to the manufacturer’s protocol. DNA was digested with Turbo DNase I (Ambion Thermo Scientific) according to the manufacturer’s instructions. Two micrograms of total RNA per sample was reverse transcribed with the High-Capacity cDNA reverse transcription kit (Thermo Scientific) using oligo dTV and random primer blend, according to manufacturer’s instructions. qRT-PCRs were run in a StepOne Plus real-time PCR system (Thermo Scientific) using a Power Sybr green PCR master mix (Thermo Scientific). Three independent biological replicates were used, and the mRNA quantity relative fold change was calculated using standard curves (74). All values were normalized to the expression of the A. fumigatus *tubA* gene. Primers are described in Table S6 at https://doi.org/10.6084/m9.figshare.12931754.v3.

### PolII chromatin immunoprecipitation coupled to DNA sequencing (PolII ChIP-Seq).

Conidia (1 × 10^7^) of the CEA17 and *ΔnsdC* strains were grown in liquid MM for 16 h at 37°C under shaking conditions. Mycelia and chromatin were prepared as previously described (75). Briefly, formaldehyde was added to the culture to a final concentration of 1% and incubated with gentle rocking for 20 min at room temperature for DNA cross-linking. After, glycine was added to a final concentration of 1 M and the culture was incubated for another 10 min to stop the reaction. Cells were filtered using Miracloth, washed twice with 100 ml of cold water, and rapidly frozen in liquid nitrogen. Thirty milligrams of the frozen mycelia were freeze-dried for at least 2 h and lysed in 800 μl of FA lysis buffer for 3 min using a Bullet blender (Next Advance). Lysis was repeated six times with a 3-min incubation on ice in between. The lysed mycelium was pelleted by centrifugation at 14,000 rpm for 15 min at 4°C and subsequently resuspended in 500 μl of FA lysis buffer and sonicated using the Qsonica Q800R at 100% amplitude with 10-s ON and 15-s OFF cycles for a total sonication time of 30 min. The arising chromatin solution was recovered by centrifugation at 14,000 rpm for 30 min at 4°C and stored at −80°C until use. Chromatin size (∼100 to 300 bp) and quality were checked on a 2% agarose gel. PolII immunoprecipitation was performed by mixing 50 μl of chromatin extract with 450 μl of FA lysis buffer and 2 μl of anti-RNA polymerase II subunit antibodies (clone 3E8; Millipore) for 1.5 h, followed by incubating the mixture with ∼15 μl packed protein A Sepharose (GE Healthcare) for another 1.5 h at room temperature on an end-to-end rotator. Subsequently, protein A Sepharose matrix was transferred to a Corning Costar SpinX centrifuge tube filter and washed. Immunoprecipitated chromatin DNA was de-cross-linked at 65°C overnight and then was purified using a Qiagen PCR cleanup purification column. Library preparation was carried out using an NEBNext Ultra II library prep kit (Illumina; catalog no. E7645L) according to the manufacturer’s protocol and barcoded adaptors as described in the work of Wong et al. (76). Libraries were checked and quantified using a DNA High Sensitivity Bioanalyzer assay (Agilent; catalog no. XF06BK50), mixed in equal molar ratio, and sequenced using the Illumina HiSeq2500 platform at the Genomics and Single Cells Analysis Core facility at the University of Macau.

Raw sequencing reads were mapped to the A. fumigatus strain Af293 reference genome (AspGD version s03-m05-r06) using Bowtie2 (v2.3.5) (77). PolII binding levels were measured by counting number of reads over coding regions and normalizing as FPKM (fragments per kilobase per million) scores. For ChIP-Seq signal data visualization purpose, aligned reads were extended to 200 bp using the ‘macs2 pileup’ command and scaled to 1 million mapped reads using the ‘macs2 bdgopt’ command from the MACS2 (v2.1.1) tool (78). UCSC Kent Utils programs (79) ‘bedSort’ and ‘bedGraphToBigWig’ were used to generate BigWig files, which were visualized in Integrative Genomics Viewer (v2.5.3) (80).

### Cell wall polysaccharide extraction and sugar quantification.

Fungal cell wall polysaccharides were extracted from 100 mg dry-frozen biomass as described previously using TCA (trichloroacetic acid) hydrolysis (81). Total carbohydrates were estimated using the phenol sulfuric method as described by Masuko et al. (82). Released sugars from hydrolysis were subsequently analyzed by high-performance liquid chromatography (HPLC) using a YoungLin YL9100 series system (YoungLin, Anyang, South Korea) equipped with a YL9170 series refractive index (RI) detector at 40°C. Samples were loaded in a Rezex ROA (Phenomenex, USA) column (300 × 7.8 mm) at 85°C and eluted with 0.05 M sulfuric acid at a flow rate of 0.5 ml/min. All sugar concentrations were expressed in millimolar (mM) using a correspondent standard curve.

### Staining for dectin-1, chitin, and other cell surface carbohydrates.

Cell wall surface polysaccharide staining was performed as described previously (83, 84). Briefly, strains were grown from 2.5 × 10^3^ spores in 200 μl of MM for 16 h at 37°C before the culture medium was removed and germlings were UV irradiated (600,000 μJ). Hyphal germlings were subsequently washed with 1× phosphate-buffered saline (PBS), and 200 μl of a blocking solution (2% [wt/vol] goat serum, 1% [wt/vol] bovine serum albumin [BSA], 0.1% [vol/vol] Triton X-100, 0.05% [vol/vol] Tween 20, 0.05% [vol/vol] sodium azide, and 0.01 M PBS) was added. Samples were incubated for 30 min at room temperature (RT). For dectin staining, 0.2 μg/ml of Fc-h-dectin-hFc was added to the UV-irradiated germlings and incubated for 1 h at RT, followed by the addition of 1:1,000 DyLight 594-conjugated, goat anti-human IgG1 for 1 h at RT. Germlings were washed with PBS, and fluorescence was read at 587-nm excitation and 615-nm emission. For chitin staining, 200 μl of a PBS solution with 10 μg/ml of calcofluor white (CFW) was added to the UV-irradiated germlings, which were incubated for 5 min at RT and washed three times with PBS before fluorescence was read at 380-nm excitation and 450-nm emission. For galactosaminogalactan (GAG), GlcN (glucosamine), and mannose staining, 200 μl of PBS supplemented with 0.1 mg/ml of either soybean agglutinin-fluorescein isothiocyanate (SBA-FITC) (Glycine max soybean lectin SBA-FITC; Bioworld; catalog no. 21761024-2), wheat germ agglutinin (WGA) (lectin-FITC L4895; Sigma), or concanavalin A (ConA; C7642; Sigma) was added to the UV-irradiated germlings for 1 h at RT. Germlings were washed with PBS, and fluorescence was read at 492-nm excitation and 517-nm emission. All experiments were performed using 12 repetitions, and fluorescence was read in a microtiter plate reader (SpectraMax i3; Molecular Devices).

### Transmission electron microscopy (TEM) analysis of cell wall.

Strains were grown statically from 1 × 10^7^ conidia at 37°C in MM for 24 h. Mycelia were harvested and immediately fixed in 0.1 M sodium phosphate buffer (pH 7.4) containing 2.5% (vol/vol) glutaraldehyde and 2% (wt/vol) paraformaldehyde for 24 h at 4°C. Samples were encapsulated in agar (2%, wt/vol) and subjected to fixation (1% OsO_4_), contrasting (1% uranyl acetate), ethanol dehydration, and a two-step infiltration process with Spurr resin (Electron Microscopy Sciences) of 16 h and 3 h at RT. Additional infiltration was provided under vacuum at RT before embedment in BEEM capsules (Electron Microscopy Sciences) and polymerization at 60°C for 72 h. Semithin (0.5-μm) survey sections were stained with toluidine blue to identify the areas of best cell density. Ultrathin sections (60 nm) were prepared and stained again with uranyl acetate (1%) and lead citrate (2%). Transmission electron microscopy (TEM) images were obtained using a Philips CM-120 electron microscope at an acceleration voltage of 120 kV using a MegaView3 camera and iTEM 5.0 software (Olympus Soft Imaging Solutions GmbH). Cell wall thicknesses of 100 sections of different germlings were measured at ×23,500 magnification, and images were analyzed with the ImageJ software (85). Statistical differences were evaluated by using one-way analysis of variance (ANOVA) and Tukey’s *post hoc* test.

### Ethics statement.

Animal experiments were performed in strict accordance with Brazilian Federal Law 11794, establishing procedures for the scientific use of animals in strict accordance with the principles outlined by the Brazilian College of Animal Experimentation (CONCEA), and the state law establishing the animal protection code of the State of São Paulo. All protocols adopted in this study were approved by the local ethics committee for animal experiments from the University of São Paulo, Campus of Ribeirão Preto (permit number 08.1.1277.53.6; Studies on the interaction of Aspergillus fumigatus with animals). All efforts were made to minimize suffering. All stressed animals were sacrificed by cervical dislocation after intraperitoneal (i.p.) injection of ketamine and xylazine.

### Murine model of pulmonary aspergillosis, lung histopathology, and fungal burden.

Outbred female mice (BALB/c strain; body weight, 20 to 22 g) were housed in vented cages containing five animals. Mice were immunosuppressed with cyclophosphamide (150 mg per kg of body weight), which was administered intraperitoneally on days −4, −1, and 2 prior to and after infection. Hydrocortisonacetate (200 mg/kg body weight) was injected subcutaneously on day −3. A. fumigatus strains were grown on YAG for 2 days prior to infection. Fresh conidia were harvested in PBS and filtered through Miracloth (Calbiochem). Conidial suspensions were spun for 5 min at 3,000 × *g*, washed three times with PBS, counted using a hemocytometer, and resuspended at a concentration of 5.0 × 10^6^ conidia/ml. The viability of the administered inoculum was determined by incubating a serial dilution of the conidia on YAG medium, at 37°C. Mice were anesthetized by halothane inhalation and infected by intranasal instillation of 1.0 × 10^5^ conidia in 20 ml of PBS. As a negative control, a group of five mice received PBS only. Mice were weighed every 24 h from the day of infection and visually inspected twice daily. The statistical significance of the comparative survival values was calculated using log rank analysis and the Prism statistical analysis package (GraphPad Software Inc.).

To investigate fungal burden in murine lungs, mice were immunosuppressed with cyclophosphamide (150 mg/kg of body weight), which was administered intraperitoneally on days −4 and −1, while hydrocortisonacetate was injected subcutaneously (200 mg/kg) on day −3. Five mice per group were intranasally inoculated with 1 × 10^6^ conidia/20 ml of suspension. A higher inoculum, in comparison to the survival experiments, was used to increase fungal DNA detection. Animals were sacrificed 72 h postinfection, and the lungs were harvested and immediately frozen in liquid nitrogen. Samples were homogenized by vortexing with glass beads for 10 min, and DNA was extracted via the phenol-chloroform method. DNA quantity and quality were assessed using a NanoDrop 2000 spectrophotometer (Thermo Scientific). At least 500 mg of total DNA from each sample was used for quantitative real-time PCRs. Specific primers were used to amplify the 18S rRNA region of A. fumigatus and an intronic region of mouse GAPDH (glyceraldehyde-3-phosphate dehydrogenase) (see Table S6 at https://doi.org/10.6084/m9.figshare.12931754.v3). Six-point standard curves were calculated using serial dilutions of gDNA from all the A. fumigatus strains used and the uninfected mouse lung. Fungal and mouse DNA quantities were obtained from the threshold cycle (*C_T_*) values from an appropriate standard curve (74). Fungal burden was determined as the ratio between picograms of fungal DNA and micrograms of mouse DNA.

For histopathology studies, the animals were also sacrificed 72 h postinfection and lungs were removed and fixed for 24 h in 3.7% formaldehyde-PBS. Samples were washed several times in 70% alcohol before dehydration in a series of alcohol solutions of increasing concentrations. Finally, samples were diaphanized in xylol and embedded in paraffin. For each sample, sequential 5-mm-thick sections were collected on glass slides and stained with Grocott’s methenamine silver (GMS) or hematoxylin and eosin (HE) stain following standard protocols. Briefly, sections were deparaffinized, oxidized with 4% chromic acid, stained with methenamine silver solution, and counterstained with picric acid. For HE staining, sections were deparaffinized and stained first with hematoxylin and then with eosin. All stained slides were immediately washed, preserved with mounting medium, and sealed with a coverslip. Microscopic analyses were done using an Axioplan 2 imaging microscope (Zeiss) at the stated magnifications under bright-field conditions.

### A. fumigatus infection in immunocompetent mice.

Eight- to 12-week-old male C57BL/6 WT mice were obtained from the specific-pathogen-free Isogenic Breeding Unit of the Department of Immunology, Institute of Biomedical Sciences, University of São Paulo. Mice were anesthetized and submitted to intratracheal (i.t.) infection as previously described (86) and further adapted to the A. fumigatus infection model (30, 87). Briefly, after intraperitoneal (i.p.) injection of ketamine and xylazine, animals were inoculated with 5 × 10^7^ CEA17 wild-type, Δ*nsdC*, or Δ*nsdC*::*nsdC*^+^ conidia, contained in 75 μl of PBS, by surgical i.t. inoculation, which allowed dispensing of the fungal cells directly into the lungs.

### Assessment of leukocyte subpopulations and flow cytometric analysis.

Lungs from A. fumigatus-inoculated mice were collected after 3 days of exposure to the microbe. To assess the leukocyte subpopulations, the lungs were removed and digested enzymatically for 40 min with collagenase (2 mg/ml) in RPMI culture medium (Sigma). Total lung leukocyte numbers were assessed with trypan blue, and viability was always >95%. For cell surface staining, leukocytes were washed and suspended at 1 × 10^6^ cells/ml in staining buffer (PBS, 2% fetal calf serum, and 0.1% NaN_3_). Fc receptors were blocked by the addition of unlabeled anti-CD16/32 (eBioscience). Leukocytes were then stained in the dark for 25 min at 4°C with the optimal dilution of each monoclonal antibody. The following were added to total leukocytes, neutrophils, and macrophages: anti-CD45, CD11b, Ly6G, Ly6C, F4/80, CD40, MHC-II, and CD86 (eBioscience or BioLegend). Cells were washed twice with staining buffer, before being fixed with 2% paraformaldehyde (PFA; Sigma). A minimum of 100,000 events was acquired on a FACSLyrics flow cytometer (BD Biosciences) using the FACSDiva software (BD Biosciences). Total leukocytes, macrophages, and neutrophils were gated as judged from forward and side light scatter. The cell surface expression of leukocyte markers was analyzed using the FlowJo software (Tree Star).

### Bone marrow-derived macrophage (BMDM) preparation, phagocytosis, and killing assay.

The BMDM preparation was performed according to the work of Marim et al. (88) with some modifications. BMDMs were prepared from C57BL/6 adult mouse femurs and tibias. These cells were cultured for 6 days in RPMI 1640 medium supplemented with 20% fetal cow serum (FCS) and 30% L-929 cell-conditioned medium. Nonadherent cells were removed, and the adherent cells (majority macrophages) were removed and washed twice with cold PBS. Cell concentration was determined using a Neubauer chamber. The phagocytic assay was performed as previously described (89) with slight modifications. In 24-well microplates at 37°C with 5% CO_2_, 1 ml of RPMI-FCS containing 1 × 10^5^ conidia (1:5 macrophage/conidium ratio) was added and incubated for 90 min. The supernatant was removed, and 0.5 ml of 3.7% formaldehyde-PBS was added. The samples were washed with ultrapure water and incubated for 20 min with 495 μl of water and 5 μl of CFW (10 mg/ml). Samples were washed and mounted on slides with 50% glycerol. Images were then acquired on an Eclipse E800 fluorescence microscope (Nikon Instruments), and the phagocytosis index was calculated counting at least 100 conidia per sample. The experiments were repeated in triplicate. To assess conidial killing, the phagocytic cells were obtained as described above. Subsequently, 1 × 10^5^ conidia (1:5 macrophage/conidium ratio) were incubated at 37°C with 5% CO_2_ for 4 h. As positive control, conidia without BMDMs were used. The 24-well microplates were centrifuged for 10 min at 3,500 rpm, and the culture supernatant was removed. Then, 100 μl of 1% Triton X-100 was added. After 10 min at room temperature, samples were removed from the microplate, washed three times with sterile distilled water, and serially diluted in PBS. The dilutions were plated and incubated at 37°C for 48 h. The percentage of conidial killing was calculated by the measurement of CFU numbers carried out after macrophage lysis, comparing CFU numbers from samples incubated with macrophages to CFU numbers from those incubated without macrophages. The experiments were repeated three times, each performed in triplicate.

### Data availability.

The transcriptional profiling for Δ*nsdC* was investigated using ChIP of RNA polymerase II coupled to DNA sequencing. Short reads were deposited in the NCBI, under GEO accession number GSE148557.
